# The construction and preliminary validation of a new Pictorial Materialism Test for 4–6-year-old children

**DOI:** 10.1371/journal.pone.0290512

**Published:** 2023-08-24

**Authors:** Agata Trzcińska, Karolina Kubicka, Wojciech Podsiadłowski

**Affiliations:** Faculty of Psychology, University of Warsaw, Warsaw, Poland; Hubei Normal University, CHINA

## Abstract

Materialistic tendencies may originate in early childhood, and previous research shows that even preschoolers differ in the degree of their materialism. The goal of this project was to develop and validate a new instrument that reliably measures materialism in preschool children (4–6 years old). A simple and universal Pictorial Materialism Test (PMT) was created and its psychometric properties were evaluated. The PMT consists of 32 items reflecting two dimensions: acquisition as the pursuit of happiness and success defined by possessions. To evaluate the psychometric properties of the PMT, a total of 204 Polish children aged 4–6 years were recruited for the study using an opportunity sampling method. To examine the reliability of the PMT, we calculated the internal consistency, confirmatory factor analysis (CFA), and test–retest intra-class correlation. To determine the convergent validity of the PMT, correlations with another measure of materialism in children (Happiness Collage) and with age were examined. The results confirmed that materialism can be reliably reported by preschool children and that the PMT has satisfactory (albeit moderate) psychometric properties. The instrument developed in this project is promising for further research because it has the potential to be used in research on materialism in preschool children around the world. In conclusion, we propose a preliminarily validated new Pictorial Materialism Test to measure general materialistic tendencies and their dimensions (possession-driven happiness and materially defined success) in 4–6-year-old children.

## Introduction

Children usually start to make comparisons with their peers when they enter preschool or kindergarten. They begin to notice that some children have more or better toys than others. At this stage, parents often feel discomfort when they go shopping with their children–children begin to ask for more and more new toys, often claiming that “everyone else has toys like that too.” However, observations from everyday life suggest that preschool children have different attitudes toward material things (like toys): it seems that material things are more important for some children than for others and some children are more inclined than others to seek happiness through the acquisition of goods. Yet there is surprisingly little research on materialism among young children. This may be because the most popular theory in the literature on materialistic tendencies in children [1] asserts that materialism appears only in school-age children. According to John’s theory [1], children begin to develop materialistic orientations in middle childhood, when they become more aware of the social aspects of consumption and the symbolic meanings of brands. They begin to associate material goods with social status, happiness, and fulfillment and to adopt materialistic values as a means to increase their personal worth, social status, or sense of belonging to a group [2, 3]. As McAlister and Cornwell note [4], John’s conclusion about the limitations of young children’s brand understanding seems convincing at first glance; however, a closer look at the evidence raises questions. The youngest children examined in the studies on brand symbolism reviewed by John were second grade students. Therefore, the studies do not contain data that allow conclusions to be drawn about preschoolers’ understanding of brand symbolism and their materialistic orientations. Moreover, given the enormous cultural changes over the past decade, especially the digital and social media revolution, today’s children are likely to develop materialistic tendencies much earlier than previously thought.

Some recent studies suggest that materialism emerges in preschool children [5]. It has been shown that children as young as 3 to 5 years of age begin to understand brand symbolism and judge their peers as being popular or unpopular and fun or boring based on the goods they own [4, 6, 7]. The use of brands to reinforce self-image indicates the early emergence of materialism in preschool children [4]. Furthermore, research by van der Meulen et al. [8] suggests that children as young as 6 years old are able to understand the symbolic meaning of material possessions. Smith et al. [9] have also shown that children as young as 5 years old have different spending and saving tendencies and can reliably express them. All in all, the above research results indicate that even preschool children differ in their materialistic tendencies and that these tendencies are probably measurable.

Nowadays, children grow up in a world full of advertisements, material objects, and ever newer toys, with consumer culture becoming an important element in their lives from a very young age. Researchers seem to agree that frequent exposure to advertising shapes materialistic attitudes in children [10, 11]. At the same time, materialism in children and adolescents has for some time been increasingly associated with a decline in emotional well-being in new generations [12–14]. Studies on adults have highlighted many negative consequences of materialism. These studies include links between materialism and mental health problems such as anxiety and depression [15], use of psychoactive substances such as alcohol and drugs [16], and egoistic attitudes and behaviors [17].

Given these findings and the importance of the problem, it seems crucial to examine materialism and its antecedents in the early stages of a person’s life. Gaining an understanding of this concept in preschool children can contribute to improved prevention and comprehension of the detrimental effects of materialism in both children and adults. However, in order to understand materialism, it is crucial to have appropriate measures. Although calls for an improved measure of materialism have been made in recent years [18], such a scale has not yet been developed for preschool children.

In this article, our objective was to develop and conduct preliminary validation of the Pictorial Materialism Test (PMT)–a new instrument for measuring materialism in children aged 4–6 years. Recognizing the lack of a reliable and child-friendly measure of materialism among preschoolers, we created a straightforward pictorial questionnaire inspired by the concept of materialism developed by Richins and Dawson [19]. The generated pictures were evaluated by competent judges and children. To validate the PMT, we carried out a study involving Polish preschoolers. Through an examination of internal consistency, factor structure, test-retest reliability, and construct validity, we conducted a preliminary validation of the PMT.

### Measurement of materialism in children

Researchers define materialism as “the importance a consumer attaches to worldly possessions” [20] and “the importance a person places on possessions and their acquisition as a necessary or desirable form of conduct to reach desired end states, including happiness” [19]. According to Richins and Dawson [19], materialism consists of three elements: (1) the centrality of acquisition–attributing the acquisition of material goods a central place in human life; (2) acquisition as the pursuit of happiness–the importance attributed to possessions in order to feel happiness and life satisfaction; and (3) success defined by possessions–the role of material goods as a determinant of life success. Richins and Dawson [19] state that materialism is primarily characterized by pursuing happiness through the acquisition of goods rather than through other measures such as relationships, experiences, or achievements. For children, Opree et al. [21] suggest that materialistic values are reflected in the importance and happiness they attach to possessions, the satisfaction they derive from acquiring new possessions, and the extent to which they like children with many possessions more than those without.

To date, there are no adequately validated instruments in the literature for measuring materialism in children under the age of 6. Existing instruments for measuring materialism in children are mostly geared toward older children and adolescents. The most popular and best validated instrument seems to be the Material Values Scale for Children (MVS-c), developed by Opree et al. [21]. It is based on the theory of Richins and Dawson [19], who distinguish three components of materialism: centrality, happiness, and success, as well as on an instrument developed by these researchers to measure materialism in adults (Material Values Scale). The Material Values Scale for Children is a self-assessment questionnaire aimed at 8–11-year-old children. This instrument has also been adapted and validated for children aged 6–8 years old by van der Meulen and coworkers [8]. Because children at this age may have reading difficulties and be suggestible, the instruments developed for this age group are administered individually and the questions are read aloud by the experimenter. Another popular measure of materialism for children is the Youth Materialism Scale by Goldberg et al. [22], developed for 9–14-year-olds.

The methods described above are questionnaire tools, but other types of methods have also been used to measure materialism in children. Chaplin and John [3, 23] proposed a very interesting measure called the Happiness Collage. In this method, children answer the question “*What makes you happy*?” by creating collages from pictures of various activities, relations, and material objects, and they can also add their own suggestions on blank cards. A child’s level of materialism is calculated based on how many pictures of material things the child has selected. This method was originally proposed for children between the ages of 8 and 18. It has also been used to examine materialism in younger children above 6 years of age [6, 8]. Watkins et al. [7] adapted this method to preschool children, taking into account for their cognitive abilities, and used it to study the impact of advertising on brand knowledge and materialism. Although the Happiness Collage was successful as a measurement of materialism in various studies, it has its drawbacks–it requires researchers performing pilot studies to update the set of pictures to represent material objects and activities that are currently attractive to children. As a result, different researchers use different versions of this instrument, which often contain very different sets of pictures. Therefore, it can be difficult to compare results between studies that use this method. Moreover, as noted by van der Meulen et al. [8], the Happiness Collage captures only one dimension of materialism–happiness derived from possessions–and does not account for other dimensions of the construct.

In addition to the methods researchers use to measure materialism in children, the literature also contains suggestions for instruments that examine similar constructs. For example, as Gąsiorowska [24] observed, estimating the size of coins can be a good way to measure the importance of money to a particular person. Previous studies have shown that individuals who ascribe a subjectively higher value to money tend to perceive it as physically larger than individuals with a lower desire for money [24–26]. In a classic study by Bruner and Goodman [27], children from poorer families overestimated the size of coins to a much greater extent than did children from richer families, probably because money is subjectively more valuable to poor families. In later studies conducted in different regions of the world, similar results have been obtained [28, 29]. Another measure developed for children and related to materialism is the Spendthrift–Tightwad Scale [9], which measures affective responses to spending and saving money in 5–10-year-old children. The authors constructed a computerized scale using photographs of plush animal toys to help children answer the questionnaire questions. It was found that children as young as 5 years old are able to reliably report their feelings about spending and saving and that these feelings are related to children’s money behavior.

In summary, there are examples of instruments in the literature that measure materialism and related constructs in older children and adolescents, but there is a lack of validated materialism measures that are appropriate for preschool-aged children.

### Present study

Materialistic tendencies may originate in early childhood, and previous research shows that even preschoolers differ in their degree of materialism [5, 7]. At the same time, previous studies have shown that materialism can have negative consequences for individuals and the economy, such as lower well-being, accumulation of debt, and deregulation of the financial industry [12, 30, 31]. For this reason, it is extremely important to identify how materialistic tendencies develop in children. However, appropriate research instruments are necessary in order to do so. Our goal, therefore, was to develop an instrument to measure materialism in children aged 4 to 6 years, based on the concept of materialism developed by Richins and Dawson [19]. We set out to develop a simple pictorial questionnaire based on a set of universal pictures that do not need to be updated regularly. We also wanted it to be possible to evaluate the created instrument’s psychometric properties and to compare the results of surveys conducted by different researchers. In this article, we describe the process of creating a new Pictorial Materialism Test (PMT) for 4–6-year-old children and the preliminary validation of its psychometric properties.

## Method

We report how we determined our sample size, how data was excluded, all manipulations, and all measures used in the study.

### Ethics statement

This research was approved by the Research Ethics Committee at the Faculty of Psychology, University of Warsaw, Opinion No. 11/06/2022. Informed consent forms for participation in the study were distributed to parents by the preschool teachers. Only children whose parents had given prior written informed consent were invited to participate in the study. The children were also asked for their consent (all children assented verbally, although some of them dropped out during the study). The authors did not have access to information that would allow identification of individual participants during or after data collection.

### Development of the scale

When we recognized the need to develop a new materialism test for preschoolers, the main idea behind the instrument was that children should be asked to choose between two situations–one related to material goods, the other related to activities or relationships that are attractive to the child. We decided to follow Richins and Dawson’s [19] definition of materialism and consider the dimensions they identified: happiness, success, and centrality. We assumed that possession-defined happiness could be measured by presenting the preschoolers images of children in materialistic and non-materialistic situations and asking them: “Which child is happier?” Similarly, we assumed possession-defined success could be measured by asking “Which child is cooler?” In constructing our instrument, we decided to exclude the dimension of centrality because we considered it too abstract a concept for children. Moreover, in the instrument format we chose, it was not possible to develop an appropriate question that preschool-aged children would understand and that would indicate the degree of centrality of acquisition.

We first conducted interviews with preschool teachers and preschool children to identify different types of situations and objects that might be attractive to 4–6-year-old children. We wanted to find some universal categories (e.g., toys in general, rather than specific toy brands). The things that are attractive to preschoolers were divided into two categories: the first included different types of activities and relationships and the second referred to material objects. In the category of activities and relationships, five situations were identified: the child paints beautifully, the mother reads a story to the child, the child has many friends, the child watches a cartoon on TV, and the child plays on the playground. The second category was related to attractive material things. Four categories were identified: the child has nice clothes, the child has a lot of toys, the child has a lot of money, and the child has gotten a present. As preschoolers typically cannot yet write or read, we decided to use a pictorial format. We asked a graphic designer to develop images showing children in the situations described above (depicting activities/relationships and material objects). All images were prepared in two versions: one in which the child in the image was a boy and one in which the child was a girl (to create two versions of the scale to reflect the child’s gender).

The next step was to check the content validity of the prepared images. We presented all 18 pictures (nine for each gender) of activities/relationships and material objects to four competent judges. All recruited judges had at least a PhD in psychology and specialized in economic psychology. Each judge rated the images on a scale from 0 –*not at all related to materialism* to 2 –*strongly related to materialism*. Table 1 shows descriptive statistics for the ratings given by the judges. It shows that the judges’ ratings for all images except those showing children watching cartoons on TV were consistent with the assumptions made when the tool was created–images showing relationships and activities were generally rated as non-materialistic, while images showing material objects were rated as associated with materialism. The ratings of images showing children watching cartoons on TV were inconclusive, so it was decided not to include these images in the test. After excluding these pictures, the degree of agreement between the competent judges (intra-class correlation) was calculated and found to be very high, ICC = 0.98, 95% CI [0.95, 0.99], *F*(15, 45) = 45.85, *p* < .001.

**Table 1:** Picture ratings by competent judges.

	Picture	Min / Max	*M*	*SD*
Pictures showing different types of activities and relationships	The boy paints beautifully	0 / 1	0.25	0.5
	The boy watches a cartoon on TV	0 / 2	1	0.82
	The boy plays on the playground	0 / 0	0	0
	Mother reads a story to the boy	0 / 0	0	0
	The boy has a lot of friends	0 / 0	0	0
	The girl paints beautifully	0 / 1	0.25	0.5
	The girl watches a cartoon on TV	0 / 2	1	0.82
	The girl plays on the playground	0 / 0	0	0
	Mother reads a story to the girl	0 / 0	0	0
	The girl has a lot of friends	0 / 0	0	0
Pictures referring to material things	The boy has a lot of money	2 / 2	2	0
	The boy has gotten a present	1 / 2	1.25	0.5
	The boy has beautiful clothes	1 / 2	1.75	0.5
	The boy has a lot of toys	2 / 2	2	0
	The girl has a lot of money	2 / 2	2	0
	The girl has gotten a present	1 / 2	1.25	0.5
	The girl has beautiful clothes	2 / 2	2	0
	The girl has a lot of toys	2 / 2	2	0

We also conducted a pilot study to investigate how children aged 4–6 years judge and understand the images (after excluding the images showing a child watching cartoons on TV). We interviewed five children (three girls and two boys, *M*_age_ = 5.30, *SD*_age_ = 0.67). Each child was presented with eight pictures (according to the child’s gender) and asked the following questions: “What is in this picture?”, “Is this boy/girl cool?” (1 –*not at all cool*, 2 –*rather uncool*, 3 –*somewhat cool*, 4 –*very cool*), “Is this boy/girl happy?” (1 –*not at all happy*, 2 –*rather unhappy*, 3 –*somewhat happy*, 4 –*very happy*), and “Why is this boy/girl happy?” From the children’s responses, it was clear that all children recognized and properly named the pictures. For all the pictures, the children rated the characters as very or somewhat cool (*M*_cool_ for each picture ranged from 3.20 to 3.40 with *SD*_cool_ = 0.45–0.55) and very or somewhat happy (*M*_happy_ for each picture ranged from 3.20 to 3.60 with *SD*_happy_ = 0.45–0.55). In their explanations of why the characters depicted in the pictures were happy, the children referred to the important elements in the picture (for example: this boy/girl is happy “because she is with her friends,” “because he likes to go down the slide,” “because she got a present,” or “because he got a banknote”), in accordance with the assumptions we made. Since all the pictures were correctly understood by the children, we decided to use them all in the Pictorial Materialism Test.

**Fig 1:**
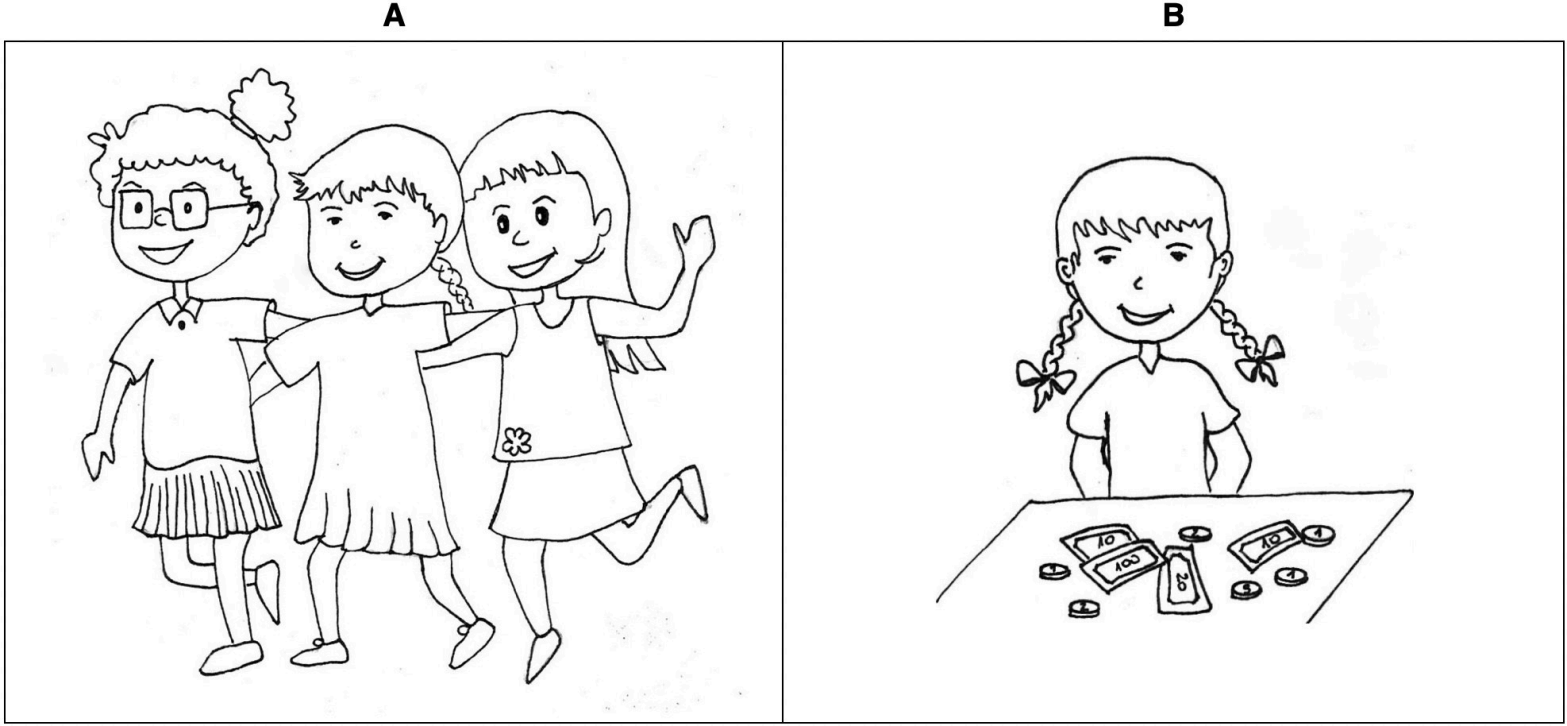
Sample item from the Pictorial Materialism Test (version for girls): “This girl has a lot of friends and this girl has a lot of money. Which girl is happier?”.

**Fig 2:**
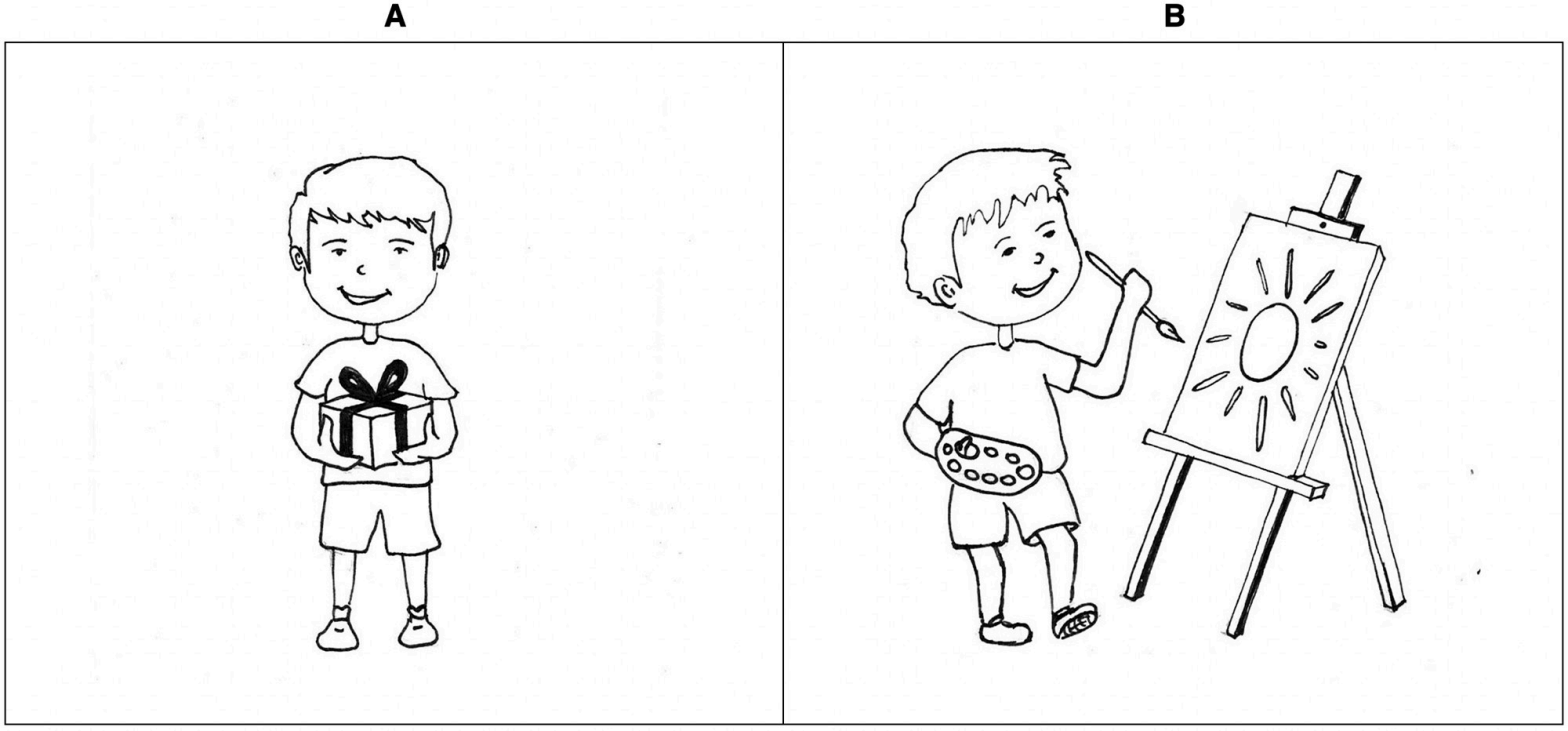
Sample item from the Pictorial Materialism Test (version for boys): “This boy has got a present and this boy paints beautifully. Which boy is cooler?”.

Pairs were created from positively verified images (four materialistic and four non-materialistic for both genders). Each image that represented an attractive material object was combined with each image that represented relationships or activities, resulting in 16 pairs. The order of the images in each pair was randomized. Consistent with the chosen methodology, which assumes measurement of two dimensions of materialism, children in the first part of the survey compared each of the 16 pairs by answering the question “Which child is happier?” (Happiness scale), while in the second part they answered the question “Which child is cooler?” (Success scale). The research procedure involved one-on-one interviews with the children, during which the investigator read the description of the pictures to the child and asked the child to choose one of the pictures. To familiarize the children with how to respond, a sample question with a pair of pictures was included at the beginning of the test. This question did not refer to material objects and was not included in the calculation of the results (“This child plays at the playground and this child’s mother reads him/her a story. Which child is happier?”). In total, the questionnaire consisted of 33 questions, 32 of which were diagnostic questions about materialism. The instrument used a forced response format–the respondent could select only one image as an answer to the question. For example, “This girl has a lot of friends and this girl has a lot of money. Which girl is happier?” (see Fig 1) or “This boy got a present and this boy paints beautifully. Which boy is cooler?” (see Fig 2). All of the images used to create the PMT can be found in S1 Appendix. The total score for materialism was the number of materialistic pictures (depicting a child with material things) selected. The score on the Happiness scale was calculated as the number of materialistic responses to the question of which child is happier, and the score on the Success scale was the number of materialistic responses to the question of which child is cooler.

In summary, in developing our new picture-based instrument to measure materialism in 4–6-year-old children, we followed Willey and Hendriks’ [32] recommendations for using picture identification tools in research with preschool children. According to these recommendations, we first had to teach the children how to respond (before the actual administration of the test), so we introduced a sample question. We used familiar terms and prepared illustrations that the children could easily recognize. The procedure involved testing each child individually and took the child’s attention into account. We also helped the children focus on specific items by, for example, printing only one pair of pictures on a page and pointing to images whose descriptions were read aloud by the experimenter. According to our pilot studies, the content of the test was age and developmentally appropriate.

### Participants

To investigate the psychometric properties of the PMT, KR-20 coefficient analyses, confirmatory factor analysis (CFA), and correlation analyses were planned. In addition, we planned to estimate test–retest reliability using intraclass correlation coefficients (ICC).

To determine the minimum sample size for the reliability test, we used the Sample Size Calculator from Arifin [33]. We expected an internal consistency coefficient of .75 with a minimum acceptable value of .65. With a power of .80 and a significance level .05 (one-tailed), the required sample size was 119. In determining the minimum sample size for confirmatory factor analysis, we relied on the results of Liang and Yang’s [34] simulation studies of models with ordered categorical data using the variance-adjusted weighted least squares estimator (WLSMV) method. The authors concluded that WLSMV estimation can be used for two-category scales (as in the Pictorial Materialism test) when the sample size is 200 or more. Therefore, our target sample size for internal reliability measures and confirmatory factor analyses was at least 200 children.

We employed an opportunity sampling method by approaching several preschools and seeking permission from the principals to conduct the study on their premises. We then invited parents and children to participate. In order to ensure a diverse sample, we specifically targeted public preschools that cater to children from different backgrounds and socio-economic statuses. The study was carried out in Warsaw, Poland, in August and September 2022. Initially, 228 children took part in our study, but 24 of them were excluded from further analyses because they did not complete the whole PMT (i.e., they wanted to stop the study at some point) or they answered all questions choosing the same option (e.g., pictures on the right-hand side of the page). Thus, 204 children aged 4–6 years (*M* = 5.15, *SD* = 0.760) were included in the final database (108 girls and 96 boys).

To establish the validity of the Pictorial Materialism Test, we planned to conduct correlational analyses. The G*Power [35] software indicated that the sample size of 64 participants would be sufficient to detect medium effects at an alpha level of 0.05 and a power of 0.80. In our study, 78 children from the entire sample completed not only the Pictorial Materialism Test but also the Happiness Collage (another measure of materialism [3, 7]). This subsample consisted of 40 girls and 38 boys aged 4–6 years (*M* = 5.42, *SD* = 0.771).

The other objective of the study was to determine the degree of test–retest reliability (two measurements) of the Pictorial Materialism Test. To determine the minimum sample size required to calculate the intraclass correlation coefficient (ICC), we used the Sample Size Calculator designed by Arifin [33]. We expected the ICC to be at least .50. A minimum sample size of 22 children would be required to achieve a statistical significance of .05 and a minimum power of .80. In our study, 27 children (16 girls and 11 boys) aged 4–5 years (*M* = 4.85, *SD* = 0.36) completed the Pictorial Materialism Test twice, two weeks apart. The age distribution of the participants is shown in Table 2.

**Table 2:** Age distribution of the participants.

Age (in years)	Boys *n* (%)	Girls *n* (%)
4	24 (11.8%)	21 (10.3%)
5	36 (17.6%)	45 (22.1%)
6	36 (17.6%)	40 (19.6%)
missing	0 (0%)	2 (1%)

### Materials and procedure

#### Pictorial Materialism Test

General Materialism and the dimensions of material Happiness and Success were measured with the new Pictorial Materialism Test, which consists of 32 diagnostic items (16 items on each subscale) described above. Children were tested individually in a quiet separate room at the preschool. For each item, an experimenter read the descriptions of two pictures to the children and asked them to choose the picture in which the child is happier (Happiness scale) or cooler (Success scale). Children were also told that there were no good or wrong answers, they can stop the game whenever they want, and that nobody will know what pictures they choose. On each question, the child scored 1 if they chose a materialistic picture (e.g., a child with many toys) and 0 if they chose another picture (referring to activities or relationships). The scores on the General Materialism scale and its subscales (Happiness and Success) were computed by calculating the sum of all corresponding questionnaire items. To assess test–retest reliability of this new measure, 27 out of the 204 children were tested again after two weeks.

#### Happiness Collage

To test the validity of our proposed measure of materialism, in addition to the Pictorial Materialism Test described above, we also assessed the level of materialism in 78 children using the Happiness Collage proposed by Chaplin and John [3, 23] and adapted for preschoolers by Watkins et al. [7]. In our study, we followed the procedure described by the latter researchers. In front of the child, the interviewer laid out and read the names of 32 colorful cards representing four themes: activities (e.g., riding a bike, drawing; seven cards total), relationships (e.g., mother, friends; nine cards), events (e.g., a trip to the zoo, birthday party; eight cards), and material objects (e.g., money, toys, clothes; eight cards). The items depicted on the cards were carefully selected beforehand to reflect the most important aspects of preschoolers’ lives. Two sets of pictures were developed to reflect the child’s gender, where appropriate. All items used in the study can be found in S2 Appendix. After the interviewer laid all the cards out in front of the child, the child was asked to select cards with activities, events, and things that make them happy. The children were allowed to select as many cards as they wanted. To calculate the level of materialism assessed by the Happiness Collage, we divided the number of material possessions selected by the total number of cards selected by a child. We expected that the Happiness Collage score would correlate positively with General Materialism measured with PMT, specifically with the Happiness subscale.

#### Gender and age

In our analyses, we also controlled for the child’s gender (dichotomous variable; girls represented by a score of 1) and age (we tested children between 4 and 6 years old).

### Analytic strategy

To validate the Pictorial Materialism Test as an instrument for measuring materialism among preschoolers, the analysis was divided into four parts. Firstly, descriptive statistics and the internal consistency of the proposed measure were examined. Secondly, confirmatory factor analysis was conducted. Thirdly, test–retest stability was measured. Finally, construct validity was examined.

In the initial analysis, the discrimination of all questions was assessed, aiming for questions where fewer than 75% of children provided the same response (e.g., the materialistic answer). Additionally, the minimum acceptable value for individual inter-item correlations was expected to be 0.15 [36, 37]. Internal consistency was evaluated using the Kuder-Richardson formula 20 (KR-20), as all items utilized a dichotomous response. A KR-20 score above 0.70 is generally considered indicative of a reasonable level of internal consistency, indicating strong item homogeneity [38].

Secondly, we conducted a confirmatory factor analysis (CFA) to determine whether our measure exhibits a two-factor or one-factor solution. Due to the dichotomous format of the PMT items, the CFA was conducted using the mean and variance-adjusted weighted least squares estimator (WLSMV) method, the analyses were performed with MPlus software version 7.2 [39]. This software provides the following fit indices for analyses with categorical indicators: normed χ^2^ (i.e., χ^2^ divided by degrees of freedom), the root-mean-square error of approximation (RMSEA), the comparative fit index (CFI), Tucker-Lewis Index (TLI), and weighted root mean square residual (WRMR). Based on the criteria listed in the literature, we assumed that the indicators of acceptable model fit were: χ^2^/df < 2, RMSEA < .08, CFI < .90, TLI < .90, and WRMR < 1 [40–42].

Third, we calculated test–retest reliability. Intra-class correlation coefficients (ICC; two-way random, absolute agreement) were calculated to test the stability of the PMT measurement over time (two measurements two weeks apart). According to Koo & Li [43], ICC values below 0.5 indicate poor agreement, while values of 0.5–0.75, 0.75–0.90, and above 0.90 indicate moderate, good, and excellent test–retest reliability, respectively.

The last step in validating the PMT was to test its validity. To measure convergent validity, all scales of the PMT were correlated with the Happiness Collage scores [3, 23]. We expected positive correlations between the PMT and Happiness Collage scores, especially for the Happiness scale of the PMT. In addition, we expected older children to exhibit lower levels of materialism in PMT. This assumption was based on previous research indicating that younger children generally derive more happiness from material goods than from experiences, but this effect changes over time: happiness derived from experiences increases with child age [44]. Also, Chan [45] found that materialism level in children was negatively correlated with age.

## Results

### Descriptive statistics and internal consistency

First, a statistical description was made for each item of the scale to check what percentage of the children gave materialistic answers. It was also checked whether there were differences between boys and girls in the frequency of materialistic responses for each question. The results are presented in Table 3. The chi-square tests showed that significant gender differences were found almost exclusively for questions where one of the options was “this child has beautiful clothes” (Q3, Q6, Q13, Q16, Q19, Q29, Q32). For these questions, girls were significantly more likely than boys to choose the materialistic answer. This result suggests that it is more important for girls to have nice clothes than for boys, which is consistent with previous research showing that appearance stereotypes are particularly prevalent in descriptions of girls [46].

**Table 3:** Frequency of materialistic responses for each question and their gender differences.

		% of materialistic answers / gender difference							
		Which child is happier? (Happiness scale)				Which child is cooler? (Success scale)			
Item		Total sample	Girls	Boys	χ^2^	Total sample	Girls	Boys	χ^2^
Q1, Q17	Has a lot of toys VS paints beautifully	32.4	28.7	36.5	1.40	35.8	30.6	41.7	2.73
Q2, Q18	Plays at the playground VS has got a present	77.0	79.6	74.0	0.92	65.2	67.6	62.5	0.58
Q3, Q19	Mom reads her/him a story VS has beautiful clothes	51.0	66.7	33.3	22.60***	52.9	61.1	43.8	6.15*
Q4, Q20	Has a lot of toys VS has a lot of friends	13.7	12.0	15.6	0.55	26.0	25.0	27.1	0.12
Q5, Q21	Paints beautifully VS has a lot of money	66.2	67.6	64.6	0.21	56.9	51.9	62.5	2.35
Q6, Q22	Has beautiful clothes VS plays at the playground	40.7	49.1	31.3	6.69*	39.2	42.6	35.4	1.10
Q7, Q23	Has a lot of toys VS mom reads her/him a story	55.9	60.2	51.0	1.72	49.5	50.9	47.9	0.18
Q8, Q24	Has a lot of friends VS has a lot of money	48.5	49.1	47.9	0.03	52.9	50.9	55.2	0.37
Q9, Q 25	Has got a present VS paints beautifully	67.2	68.5	65.6	0.19	55.4	50.9	60.4	1.85
Q10, Q26	Plays at the playground VS has a lot of toys	47.5	53.7	40.6	3.49	50.0	51.9	47.9	0.32
Q11, Q27	Mom reads her/him a story VS has a lot of money	57.8	64.8	50.0	4.57*	55.9	50.9	61.5	2.29
Q12, Q28	Has got a present VS has a lot of friends	45.6	43.5	47.9	0.40	46.6	48.1	44.8	0.23
Q13, Q29	Paints beautifully VS has beautiful clothes	44.1	55.6	31.3	12.18***	48.0	57.4	37.5	8.07**
Q14, Q30	Has a lot of money VS plays at the playground	52.9	53.7	52.1	0.05	58.3	52.8	64.6	2.91
Q15, Q31	Has got a present VS mom reads her/him a story	55.9	56.5	55.2	0.03	62.7	64.8	60.4	0.42
Q16, Q32	Has a lot of friends VS has beautiful clothes	34.3	40.7	27.1	4.21*	44.6	56.5	31.3	13.10***

*p < .05,

**p < .01,

***p < .001

The descriptive statistics presented in Table 3 show the percentage of children tested who answered materialistically for each item. From these results, it can be concluded that most questions discriminated well (most *p* values for the entire sample ranged from 0.25 to 0.75). However, for two questions, the vast majority of children (more than 75%) gave the same answers: for Q2, 77% of children gave materialistic answers and for Q4, 86.3% of children gave non-materialistic answers. Given the low level of variability in these questions, it is worth considering whether it is appropriate to retain them in the PMT. However, Nunnally [47] points out that selecting items based on *p* values alone is not a sufficient strategy. Therefore, further analyses also examined item-total correlations and changes in the KR-20 coefficient due to the removal of individual items. Detailed results can be found in Table 4. The literature suggests that the minimum acceptable value for individual inter-item correlations is 0.15 [36, 37], and in our study two items did not reach this minimum (items Q2 and Q4).

**Table 4:** Item-total correlations of PMT items for one-factor and two-factor versions.

N	One factor: General Materialism (Q1–Q32)		Two factors analyzed separately: Happiness (Q1–Q16) and Success (Q17–Q32)	
Item	Corrected item-total correlation	KR-20 if item deleted ^a^	Corrected item-subscale correlation	KR-20 if item deleted ^a^
Q1	.15	.807	.15	.685
Q2	.08	.808	.11	.688
Q3	.40	.797	.39	.656
Q4	.09	.807	.04	.692
Q5	.37	.798	.32	.665
Q6	.25	.803	.27	.672
Q7	.14	.807	.17	.684
Q8	.43	.796	.42	.653
Q9	.16	.806	.15	.685
Q10	.26	.803	.30	.667
Q11	.42	.796	.46	.647
Q12	.35	.799	.29	.669
Q13	.39	.797	.40	.655
Q14	.34	.799	.31	.667
Q15	.36	.799	.36	.660
Q16	.42	.797	.31	.666
Q17	.21	.804	.15	.721
Q18	.38	.798	.36	.698
Q19	.27	.802	.29	.706
Q20	.23	.803	.21	.713
Q21	.39	.797	.37	.697
Q22	.30	.801	.29	.706
Q23	.28	.802	.25	.710
Q24	.42	.796	.42	.692
Q25	.33	.800	.36	.698
Q26	.27	.802	.26	.709
Q27	.35	.799	.34	.700
Q28	.25	.803	.26	.709
Q29	.29	.801	.30	.705
Q30	.36	.798	.39	.696
Q31	.35	.799	.36	.699
Q32	.38	.798	.36	.698

^a^ Indicates internal consistency (KR-20) of remaining items if the elected item was deleted from the total score.

The internal reliability of the whole materialism scale was good (KR-20 = .806), but further analysis showed that deletion of some questions (items Q1, Q2, Q4, and Q7) would slightly increase the KR-20 coefficient. The reliability of the Happiness scale was not perfect, but was acceptable [48] at KR-20 = .684. Again, deletion of some questions would increase the reliability coefficient (Q1, Q2, Q4, and Q9). For the Success scale, the KR-20 coefficient was .717 and removing one item (Q17) could slightly increase reliability.

Looking at the results described above in terms of *p* values, item-total correlations, and possible changes in the KR-20 coefficient due to item removal, we found that two questions (Q2 and Q4) were the weakest, taking into account all the criteria mentioned above. Therefore, it was decided to perform the reliability analysis without these questions: KR-20 for General Materialism was then .809, for the Happiness scale was .695, and for the Success scale the value remained the same as there were no items removed (.717). These results show that the exclusion of Q2 and Q4 improved the internal reliability of the scale. However, researchers point out that in the initial stages of research instrument development, it is worthwhile to be cautious when removing items from scales [49] because individual items may have different response distributions in different samples. Therefore, in further analyses, it was decided not to completely remove items Q2 and Q4 from the PMT, but to present results for two versions of the scale–one containing all questions (32 items) and one from which two questions were removed (30 items).

The next step in the analysis was to calculate descriptive statistics for the children’s summed scores on the General Materialism scale and the two subscales, Happiness and Success. Table 5 shows means and standard deviations and the test for gender differences for all scales (both the full scale with 32 items and the scales without Q2 and Q4). As shown in Table 5, girls tended to be more materialistic than boys on the Happiness scale. This effect was mainly due to the fact that, as shown above (Table 3), girls selected pictures with nice clothes more often than did boys.

**Table 5:** Descriptive statistics for the General Materialism scale and the happiness and success subscales as well as the results of the Happiness Collage with analysis of gender differences.

Scale	Total sample			Boys	Girls	*t*	Cohen’s *d*
	*M* (*SD*)	Skewness (*SE*)	Kurtosis (*SE*)	*M* (*SD*)	*M* (*SD*)		
General Materialism (32 items)	15.91 (5.86)	-.17 (.17)	.24 (34)	15.08 (5.49)	16.64 (6.11)	1.90	.27
General Materialism (30 items)	15.00 (5.77)	-.17 (.17)	.20 (.34)	14.19 (5.42)	15.77 (6.00)	1.91	.27
Happiness (16 items)	7.91 (3.20)	-.11 (.17)	.08 (.34)	7.24 (3.06)	8.50 (3.22)	2.86**	.40
Happiness (14 items)	7.00 (3.09)	-.07 (.17)	-.11 (.34)	6.34 (2.97)	7.58 (3.09)	2.92**	.41
Success (16 items)	8.00 (3.43)	-.33 (.17)	.04 (.34)	7.84 (3.17)	8.14 (3.66)	0.61	.09
Happiness Collage	0.30 (0.15)	.76 (.27)	1.06 (.54)	0.32 (0.13)	0.28 (0.17)	-1.28	-.29

*p < .05,

**p < .01,

***p < .001

**Fig 3:**
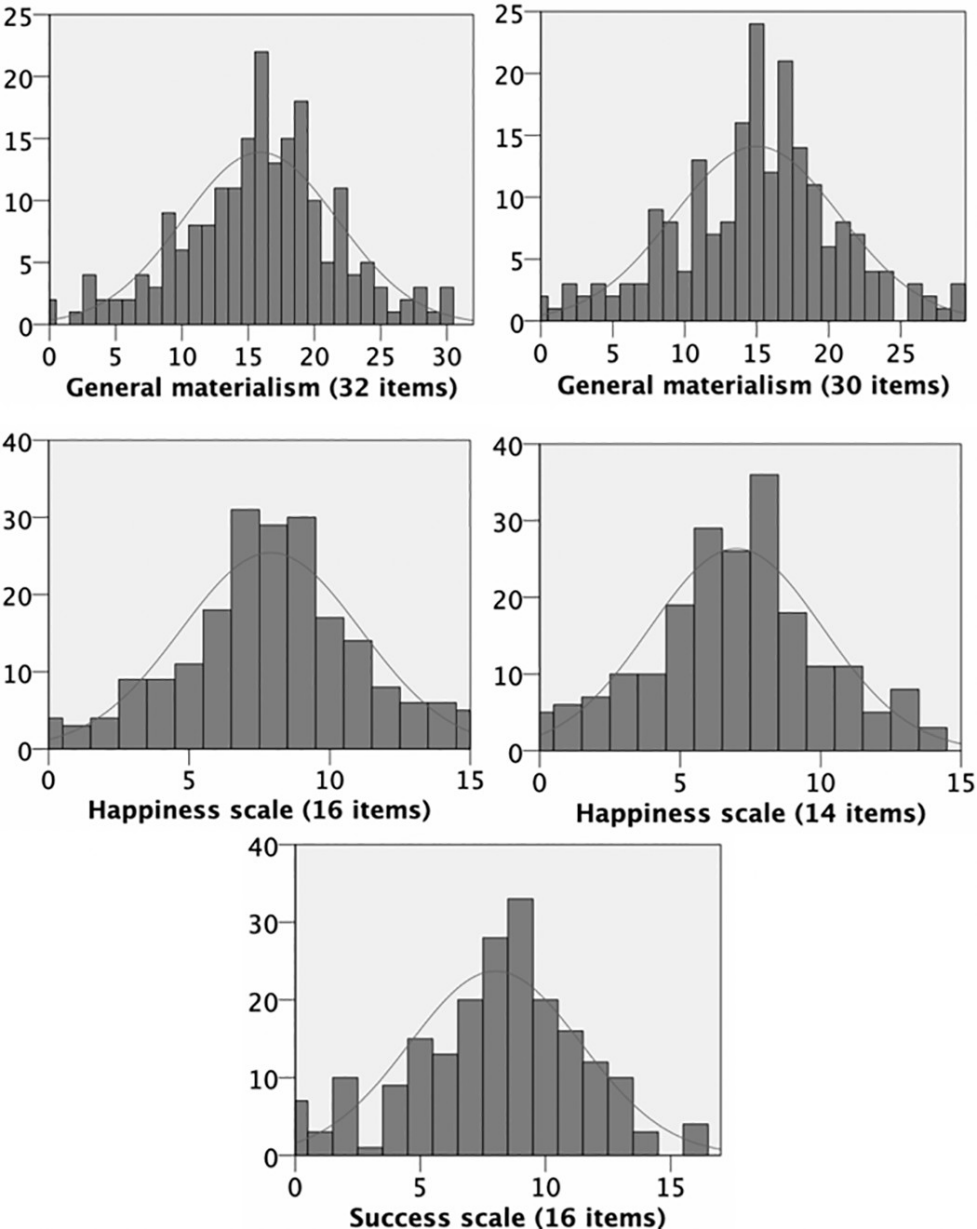
Distributions of the answers (frequencies) for each scale of the questionnaire (in the complete and in the short version).

A combination of formal normality tests, assessment of skewness and kurtosis (Table 5), and visual inspection (Fig 3) was used to assess whether a normal distribution of responses could be assumed for each scale [50]. Following Kim’s [50] rules for assessing normality distribution in medium-sized samples (50 < *N* < 300), the data were found to meet the normality assumption for all scales, although the formal normality tests (Kolmogorov–Smirnov and Shapiro–Wilk) were significant.

### Confirmatory factor analysis

The construction of the PMT assumed an a priori factorial structure (two subscales of General Materialism), so we performed confirmatory factor analysis. The purpose of this analysis was to examine the internal structural validity of the Pictorial Materialism Test and to empirically compare several models of materialism in preschool children. The following models were tested: one factor (General Materialism, Model 1), two correlated factors (Happiness and Success, Model 2), and a higher-order model (higher-order factor: General Materialism, lower-order factors: Happiness and Success, Model 3). We also tested analogous models 4, 5, and 6 (see Table 6), but without Q2 and Q4. In all models, the shared measurement error was controlled by correlating error terms for items with the same pairs of pictures but measuring different aspects of materialism: happiness and success (eQ1 with eQ17, eQ2 with eQ18, eQ3 with eQ19, etc.). It is worth noting that researchers point out that there are several problems with correlating residuals based on post-hoc modification indices [51, 52], but in our study we had a priori good reasons to correlate the error terms because each pair of pictures was presented to the child twice: once with the question “Which child is happier?” (Happiness scale) and then with the question “Which child is cooler?” (Success scale). As postulated by previous researchers [51], it is reasonable to allow for correlated residuals associated with indicators that share components (as it is in PMT).

**Table 6:** Fit indices for investigated models.

	χ^2^	df	*p*	χ^2^/df	RMSEA [95%CI]	CFI	TLI	WRMR
**Model 1** (one factor, 32 items)	567.73	448	.0001	1.27	.036 [.026–.045]	.869	.855	1.022
**Model 2** (two factors, 32 items)	543.13	447	.0012	1.22	.032 [.021–.042]	.895	.884	0.988
**Model 3** (higher-order 32 items)	560.78	448	.0001	1.25	.035 [.025–.044]	.877	.864	1.017
**Model 4** (one factor, 30 items)	506.89	391	.0001	1.30	.038 [.028–.047]	.875	.861	1.022
**Model 5** (two factors, 30 items)	481.23	390	.0011	1.23	.034 [.022–.044]	.902	.890	0.983
**Model 6** (higher-order, 30 items)	499.62	391	.0002	1.28	.037 [.026–.046]	.883	.870	1.016

**Fig 4:**
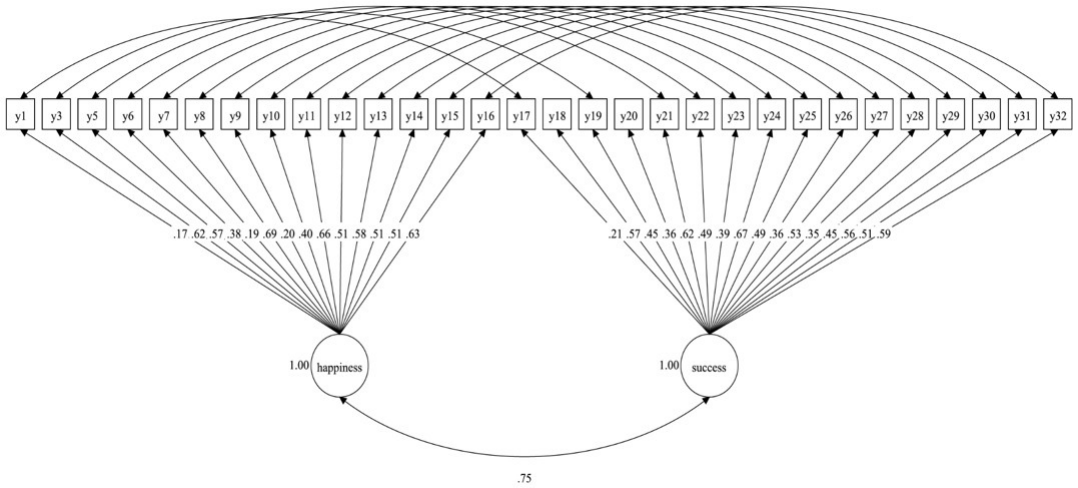
The two-factor model of the 30-item Pictorial Materialism Test with happiness and success as correlated factors (Model 5). All factor loadings are standardized.

The confirmatory factor analysis results obtained for models 1, 2, and 3 (with all 32 questions) showed that although RMSEA and χ^2^/df were acceptable, the values of CFI and TLI were slightly too low, and WRMR was acceptable only for Model 2 (see Table 6). In addition, in all of these models, all items except Q2 and Q4 had significant (*p* < .05) positive factor loadings on their designated factors. Therefore, it was reasonable to perform confirmatory factor analyses after excluding items Q2 and Q4, especially since these same questions were problematic in previous analyses (reliability and item-total correlations). These analyses are presented in Table 6 as Models 4, 5, and 6. Although the fit indices were relatively similar between these models, we selected the model with the highest CFI and TFI and the lowest WRMR (Model 5) as the best model (see Fig 4). This model had acceptable fit to the observed data for most fit indices (χ^2^/df < 2, RMSEA < .08, CFI < .90, WRMR < 1); only the TFI was slightly below the acceptable value (.90). Nevertheless, it is worth noting that CFI and TLI tend to reject models with low factor loadings for categorical indicators [53]. In Model 5 (as in Models 4 and 6), all factor loadings were significant but relatively low. Therefore, we relaxed our criteria for acceptable model fit somewhat and accepted a TLI of .89. In addition, the χ^2^ statistics were significant for all models (indicating poor model fit), but according to Li [54], models with categorical indicators are rejected more often than expected with a relatively small sample size (*N* = 200) using the adjusted chi-squared test statistics. Given this, we followed this author’s suggestion to consider other fit indices (such as RMSEA and CFI).

### Test–retest reliability

Results for the General Materialism scale (32 items) indicated good test–retest reliability–ICC = .752, *F*(26,26) = 4.031, *p* < .001. The stabilities of the Happiness and Success scales were moderate–ICC = .536, *F*(26,26) = 2.157, *p* = .028 and ICC = .566, *F*(26,26) = 2.306, *p* = .019, respectively. We also analyzed test–retest reliability after excluding items Q2 and Q4. For the General Materialism scale (30 items), we found ICC = .731, *F*(26,26) = 3.722, *p* < .001, indicating moderate agreement. For the shortened Happiness scale (14 items), we found ICC = .513, *F*(26,26) = 2.055, *p* = .036, also indicating moderate test–retest reliability.

### Construct validity

As expected, Happiness Collage results correlated positively with General Materialism from PMT (measured with 32 items) and specifically with Happiness Scale scores (measured by both 16 and 14 items). The correlation between Happiness Collage scores and PMT Success Scale scores was not significant, demonstrating the discriminant validity of this subscale. Detailed results are presented in Table 7.

**Table 7:** Correlations between PMT results and Happiness Collage scores and child age.

	General materialism		Happiness		Success
	32 items	30 items	16 items	14 items	16 items
**Happiness Collage**	.227*	.222	.267*	.260*	.141
**Age**	-.306***	-.308***	-.260***	-.263***	-.280***

*p < .05,

**p < .01,

***p < .001

In addition, we expected older children to exhibit lower levels of materialism on the PMT. This assumption was based on previous research indicating that younger children generally derive more happiness from material goods than from experiences, but this effect changes over time: happiness derived from experiences increases with child age [44]. Also, Chan [45] found that materialism level in children was negatively correlated with age. Therefore, we correlated the results of PMT with the age of the children, and the results are shown in Table 7. As expected, all correlations were negative and significant: older preschoolers were less materialistic than younger ones.

## Discussion

Our main goal was to develop an instrument that measures the materialism of preschool children (4–6 years old) in a reliable and valid way. A simple and universal Pictorial Materialism Test (PMT) based on the concept of materialism of Richins and Dawson [19] was developed and its psychometric properties were tested. To create the PMT, we collaborated with preschool teachers and children as well as a graphic designer to depict various material objects, activities, events, and relationships that are attractive to children. The images created were evaluated by competent judges and by preschoolers. In the PMT, children are presented with pairs of pictures, one showing a child in various relationships or engaged in different activities and the other showing a child with attractive material objects. The children’s task is to answer the question “Which child is happier?” or “Which child is cooler?” for each pair of pictures. According to the theoretical assumptions adopted, these questions should reflect the two dimensions of materialism distinguished by Richins and Dawson [19]–that is, acquisition as the pursuit of happiness and success defined by possessions. In the tool construction phase, we omitted the dimension of the centrality of acquisition, the third element of materialism elaborated by Richins and Dawson [19], because we found this concept too abstract for preschoolers. Finally, an instrument consisting of 32 questions was developed, which, according to the theoretical assumptions, forms two subscales of materialism: Happiness and Success. Scores for each scale (Total Materialism and its subscales Happiness and Success) were calculated as the sum of the materialistic responses on these scales–the number of pictures with material objects selected by the child.

In analyzing the psychometric properties of the PMT, we first checked that all questions had good discrimination. Then, item-total correlations and changes in the Kuder–Richardson reliability coefficient due to the deletion of individual items were analyzed. The results showed that most questions performed quite well, with the exception of Q2 and Q4. As it is worthwhile to be cautious in eliminating questions from the scale in the early stages of developing new measurement instruments [49], we conducted further psychometric analyses for both the scale with all 32 items and the shortened scale with 30 items. The analysis of KR-20 coefficients showed that the PMT scales are characterized by satisfactory reliability. The analysis of the distribution of the results showed that the children’s scores on the General Materialism scale and the Happiness and Success subscales were close to the normal distribution. An interesting finding was that girls scored higher on the materialism scales measured by our PMT, while there were no gender differences in the level of materialism measured with the Happiness Collage. In previous studies, the results for gender differences in materialism were also mixed. Some studies found that boys were more materialistic than girls [1, 17], while others observed no gender differences [8]. In our study, we identified that gender differences in General Materialism scores in PMT were primarily due to gender differences in responses to questions about clothing. This result, in conjunction with the findings of other researchers, suggests that the differences in materialism tendencies between boys and girls found in various studies are largely dependent on what specific material goods children are asked about in the materialism measurement instrument used. For example, in the Happiness Collage used in the study of van der Meulen et al. [8], girls had lower scores on the materialism scale, which the researchers explained by the fact that many of the collage items used in their study were more popular among boys than girls (e.g., items related to technology). Thus, both in the PMT we created and in measuring materialism with other instruments, one should be particularly cautious when attempting to draw conclusions about gender differences in materialism in children.

The next stage of the initial PMT validation was confirmatory factor analysis. The model assuming two correlated factors (Happiness and Success, after Q2 and Q4 were removed) proved to be the best fitting among the various models tested, confirming that materialism is not a unitary construct even among preschoolers.

Further validation analyses showed moderate test–retest reliability and confirmed the construct validity of the new PMT. The Happiness scale of the PMT correlated positively and significantly with the Happiness Collage scores, whereas the Success scale scores did not. This is another argument for the two-factor structure of our instrument, because the Happiness Collage addresses acquisition as the pursuit of happiness, but not success defined by possessions, as children answer the question “What makes you happy?” in this method. Similar results were obtained by van der Muellen et al. [8] in their study with 6–8–year-old children. They found that the collage measure of materialism was significantly associated with material centrality and material happiness but not with material success, although the correlations they identified were moderate rather than strong, as in our study.

Furthermore, as expected, both scales of the PMT and General Materialism score correlated negatively with age. This result is consistent with previous reports showing that younger children derive more satisfaction from material objects than from experiences [44] and that children aged 6–7 years score significantly higher on the materialism test than their older peers [45]. Interestingly, Chaplin and John [3] have shown that materialism increases in early adolescence because self-esteem decreases during this time. Our instrument allows us to examine earlier age differences in materialistic attitudes (among preschoolers), although the reasons for these changes require further research.

### Theoretical contributions

This study contributes to the theoretical understanding of materialism by focusing specifically on preschool-aged children. This expands the existing literature on materialism, which has predominantly focused on older children, adolescents, and adults [8, 19, 21, 22]. We propose a new Pictorial Materialism Test designed to measure general materialistic tendencies and their dimensions (possession-driven happiness and materially defined success) in 4–6-year-old children. Consistent with current research, we have provided further evidence that materialism emerges early in a child’s life [5, 7]. We confirmed that materialism can be reliably reported by preschool children, and we conducted preliminary analyses of the psychometric properties of the PMT. To our knowledge, the instrument we developed is the first test to measure materialism in children aged 4–6 years that can examine not only possession-related happiness (as in the Happiness Collage), but also perceptions of success through the lens of material goods. The study confirms the presence of two distinct dimensions of materialism, namely happiness and success, among preschool children. This finding aligns with the conceptualization proposed by Richins and Dawson [19] and highlights the importance of considering both dimensions when studying materialistic attitudes and behaviors in young children. It adds to the theoretical understanding of materialism by demonstrating that these dimensions are present and measurable even at an early age.

Moreover, by examining materialistic attitudes in preschool children, this study provides a developmental perspective on materialism. It reveals age-related differences, with younger children showing a stronger inclination towards material objects. This finding contributes to theories of economic socialization and supports the notion that materialistic tendencies may change as children grow older and experience developmental shifts in their sources of satisfaction [44].

### Practical implications

The development of the PMT offers a valuable tool for early detection of materialistic attitudes in young children. This may have practical implications for interventions and preventive measures, as early identification of materialistic tendencies can enable targeted interventions to promote alternative values, attitudes, and behaviors related to material possessions. Addressing materialism at an early stage may mitigate potential negative consequences in later stages of development [12, 15, 16, 30, 31].

Another practical implication is the ability to reliably and effectively assess materialistic tendencies in young children in different countries and compare results across cultures. The instrument developed in this project is universal: the images used do not refer to specific brands and do not refer to current fashions for different types of toys. Therefore, it can potentially be used in research on materialism in preschool children around the world.

### Limitations and future directions

While the PMT that we have developed and preliminarily validated seems promising for further research, it has some limitations. First, confirmation of the final factor structure requires replication in subsequent studies. Although in our study the best-fitting model for the data was a model with two correlated factors (Happiness and Success), we encourage other researchers to consider and compare different models (one-factor, two-factor, and higher-order factor models) in their research with the PMT. In addition, further validation studies conducted on large research samples should lead to a final decision regarding which PMT questions should remain in the final version of the PMT (i.e., whether the 32- or 30-item version is better). In addition, in this study, we evaluated PMT items liberally because we did not want to make exclusions based on a single study sample. However, it is worth noting that some questions had relatively low factor loadings, so perhaps they should not be included in the next versions of the instrument. For now, however, it seems best to include all 32 items in future research studies with the PMT and to conduct further validation analyses.

This study was conducted in a specific cultural context and focused on preschool-aged children in Poland. Therefore, caution should be exercised in generalizing the results to other cultures. First, children in different countries and cultures may have different conceptions of money and material goods. Previous studies have shown that Polish children aged 4–6 years know that money can buy things and that money is an important symbol for them [55]. However, this may not be true for preschoolers from other cultures, for example, those where children do not have the opportunity to observe or participate in economic transactions [56, 57]. Second, children learn about money and material goods by observing adults, other children, and also through the media. Accordingly, the way children interact with money and material goods may vary by culture. For example, Clarke and Micken [58] have shown that materialistic societies such as France pay particular attention to product design, whereas collective societies such as Mexico focus on the functional properties of the product. Therefore, it is important to conduct future PMT validation studies in other cultures.

The instrument we developed to measure materialism in children aged 4–6 years needs further validation studies, but it has great potential for opening the door to important research on the determinants of materialism in young children. Although there are many studies in the literature on the origins of materialism and Richins [59] has proposed a comprehensive developmental model of the emergence of materialism in children, our conclusions pertain primarily to school-age children and adolescents. To date, there have been very few studies with preschool children, likely due (in part) to the limited availability of instruments appropriate for this age group. Given that materialism can develop in the early years of a child’s life and has a variety of negative consequences in subsequent years (see [18] for a review), it is worthwhile to analyze its early origins and ways to mitigate it. For example, it is worth examining the relationship between materialism in preschool children and (1) their parents’ materialistic aspirations (for adolescents, see [60, 61]), (2) their use of various media (see [62, 63] results for 8–17-year-olds), and (3) family socio-economic status (see [64–66] for 8–17-year-olds). It would also be worthwhile to analyze in future studies the relationships between materialism in preschool children and various psychological variables, such as self-esteem (see [3] for 8–16-year-olds), life satisfaction (see [67] for 8–11-year-olds), or relationships with peers and parents (see [68] for 12–18-year-olds).

## Supporting information

S1 Appendix Images used in the Pictorial Materialism Test. (DOCX)

S2 Appendix Happiness Collage items. (DOCX)
