# Supplementary figures and images for: The construction and preliminary validation of a new Pictorial Materialism Test for 4–6-year-old children
Source: PLoS One. 2023 Aug 24;18(8):e0290512. doi: 10.1371/journal.pone.0290512 (PMC10449474; doi:10.1371/journal.pone.0290512)

**S1 Appendix. Images Used in the Pictorial Materialism Test**

| **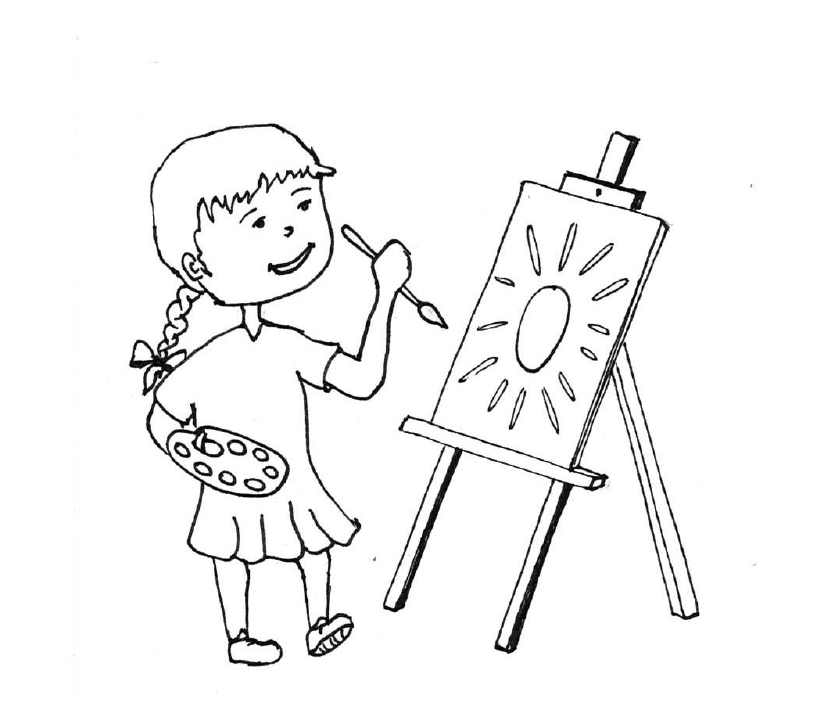** | **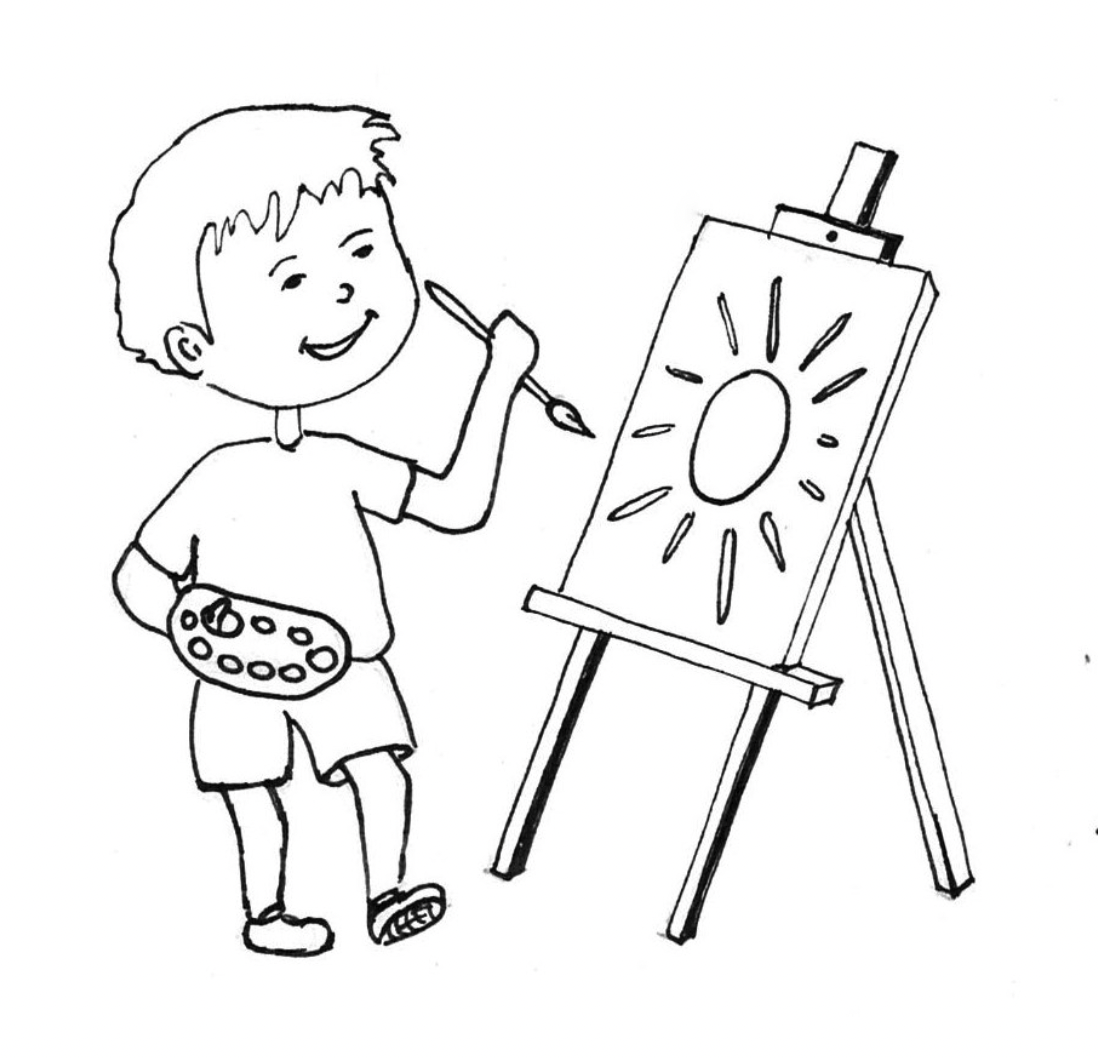** |
| --- | --- |
| **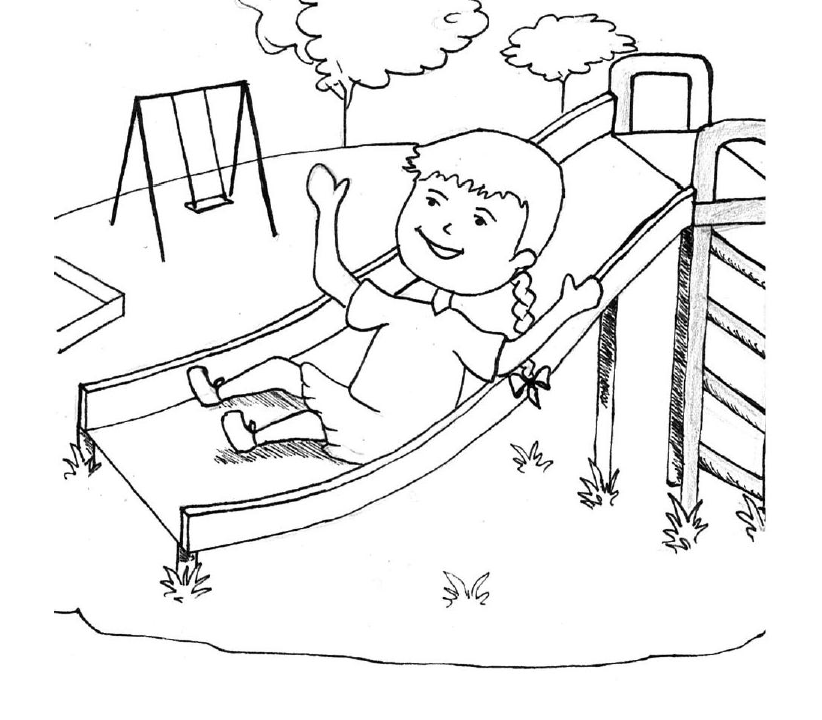** | **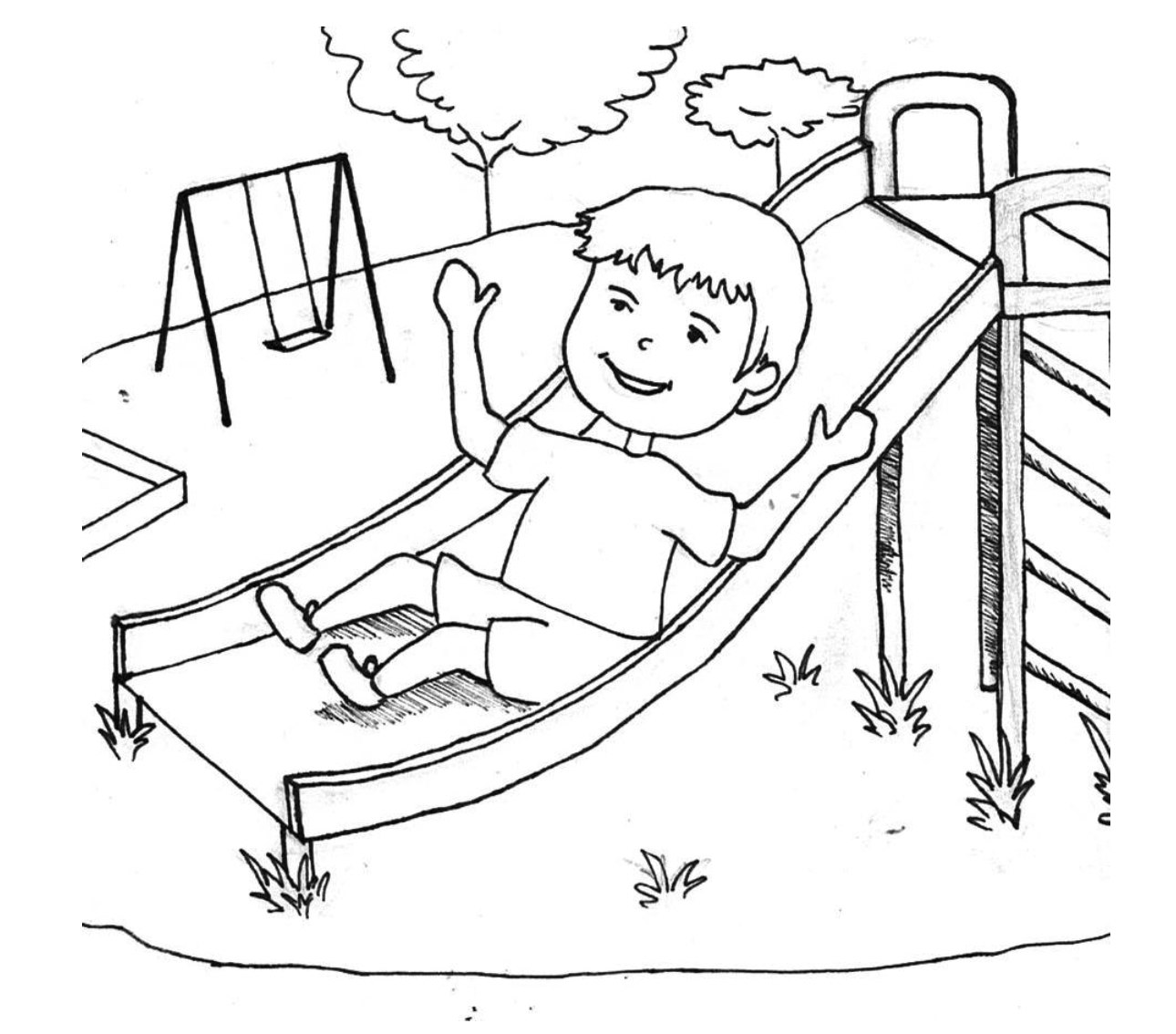** |
| **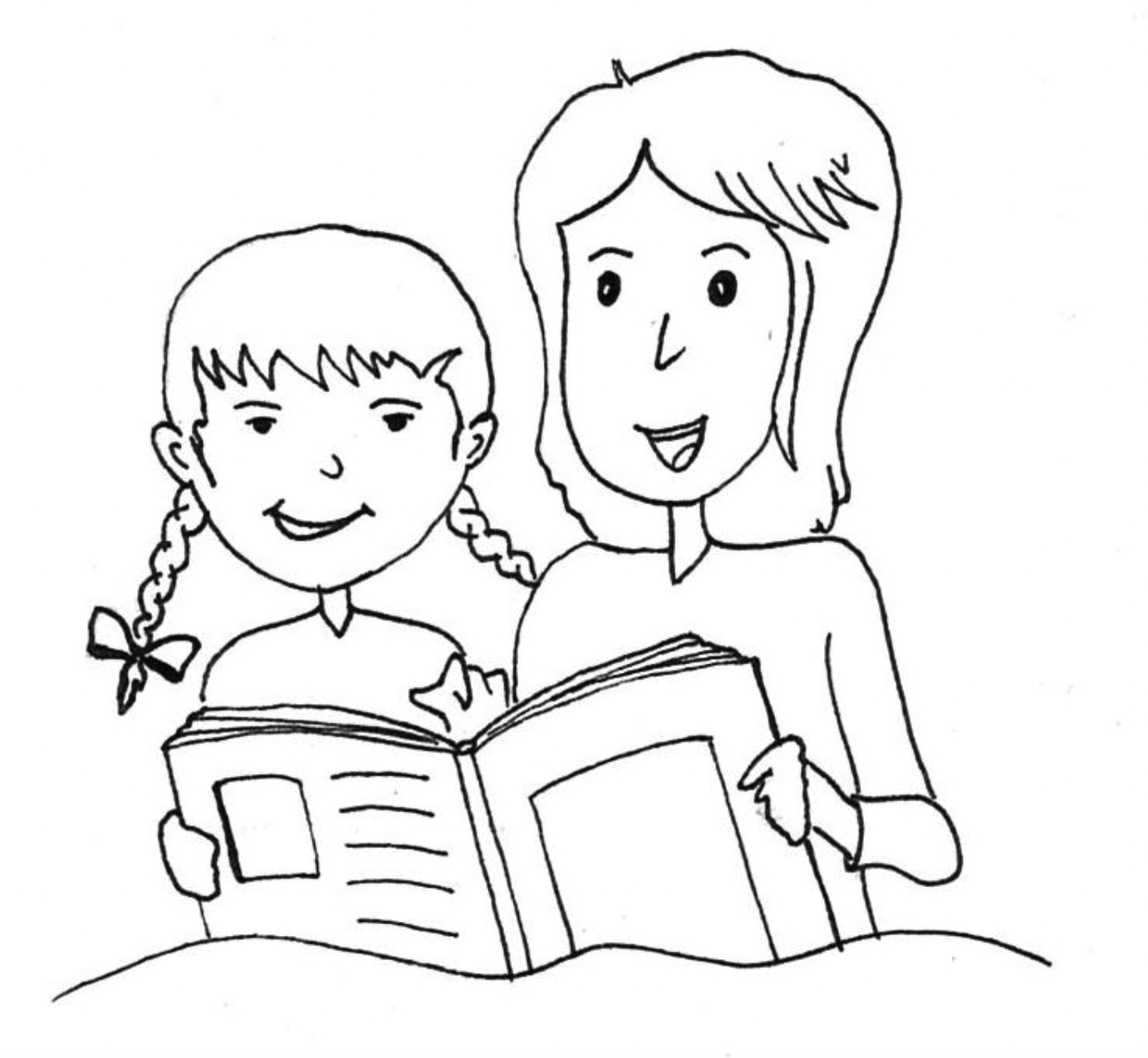** | **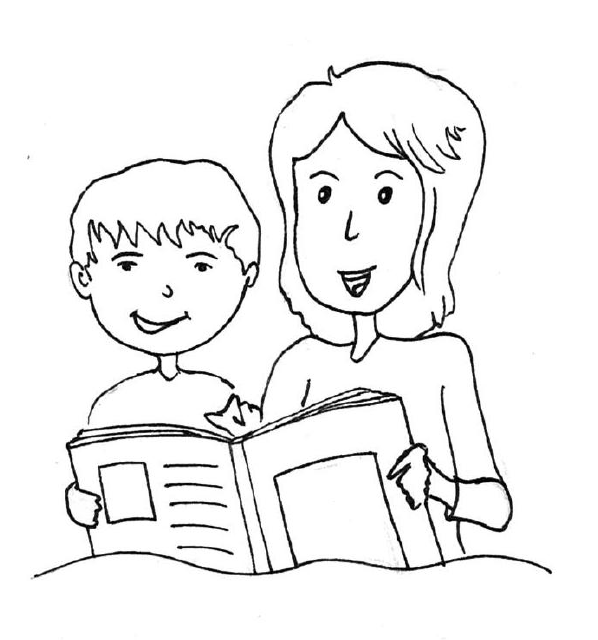** |
| **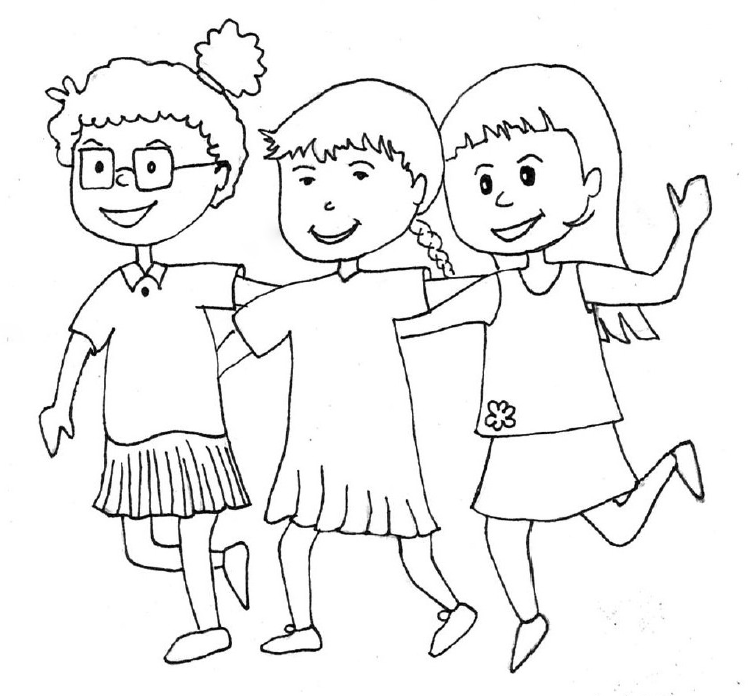** | **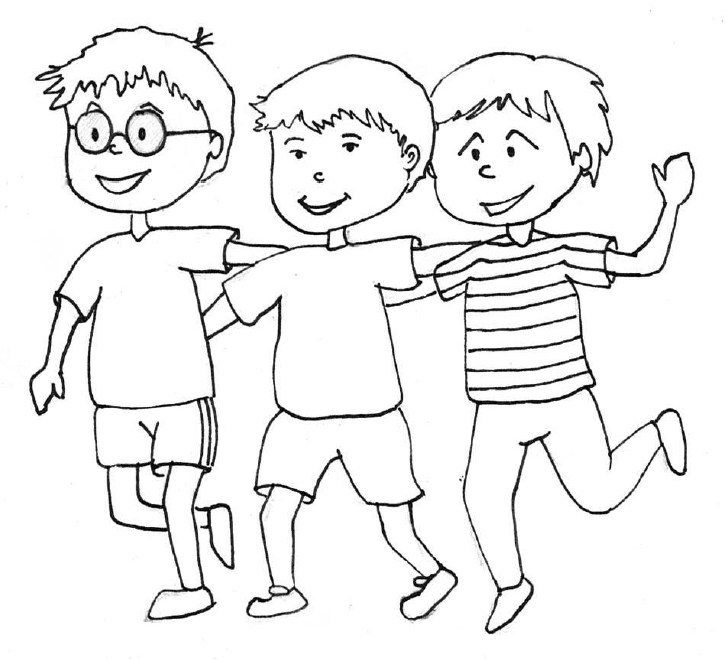** |
| **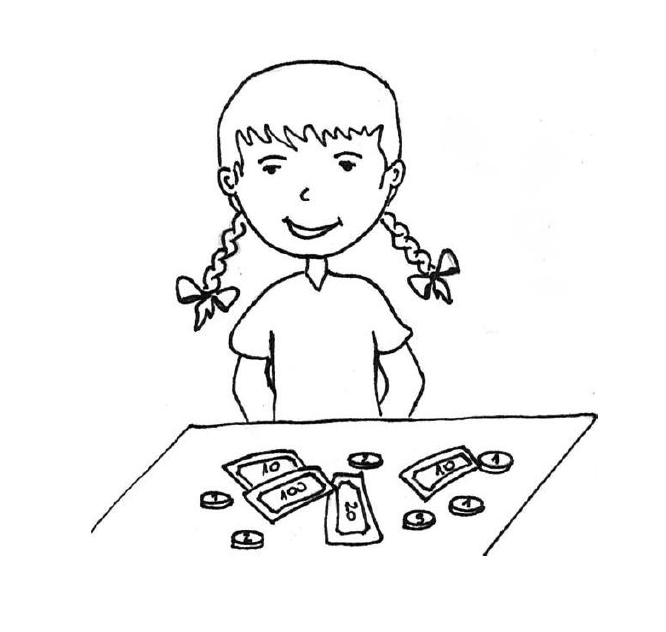** | **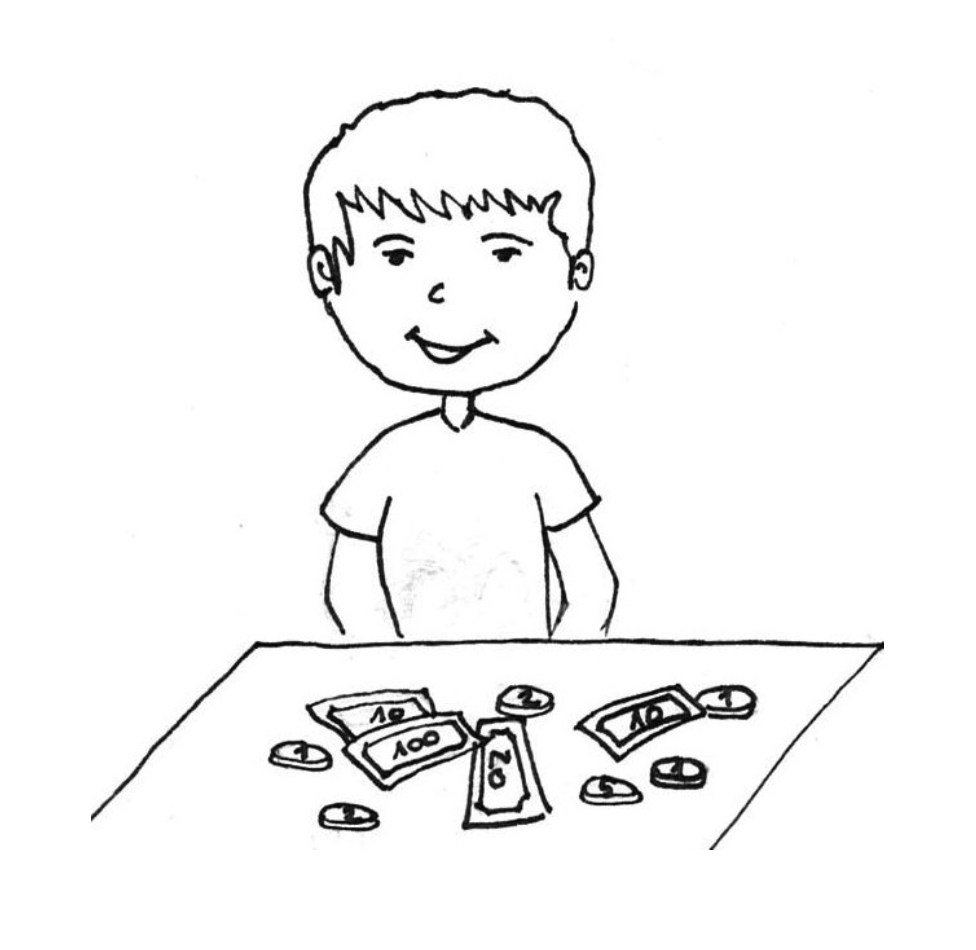** |
| **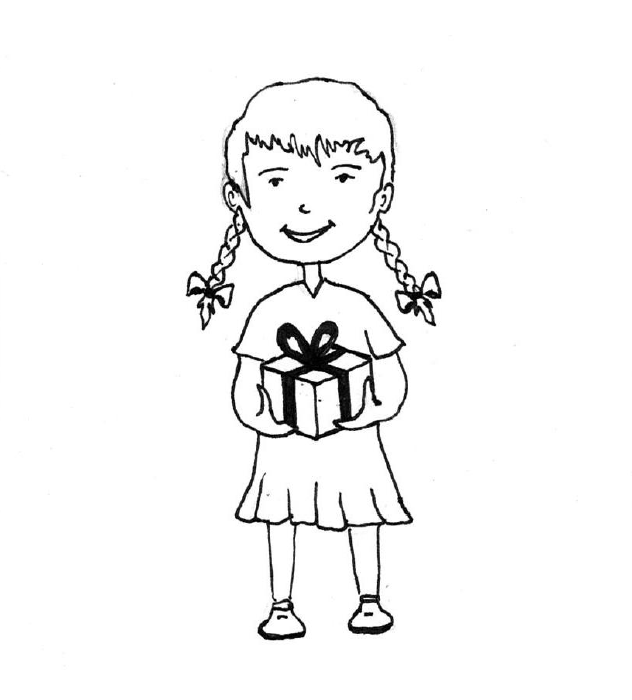** | **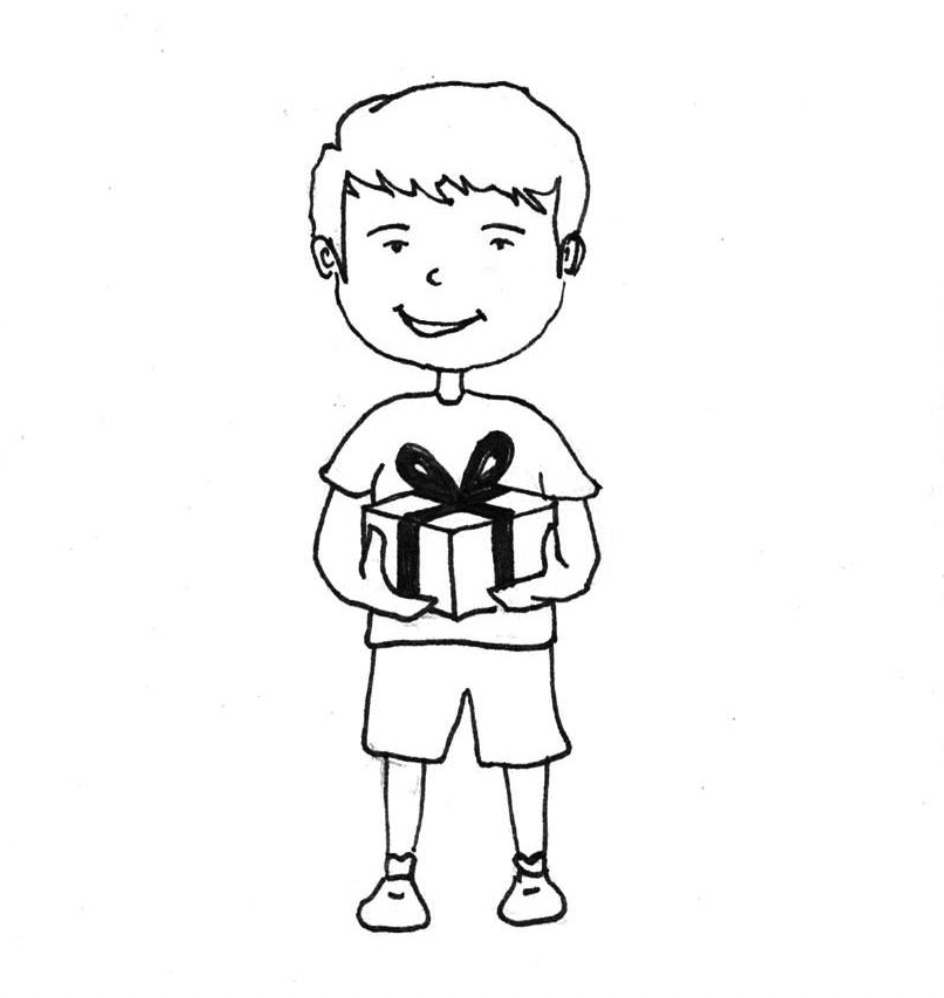** |
| **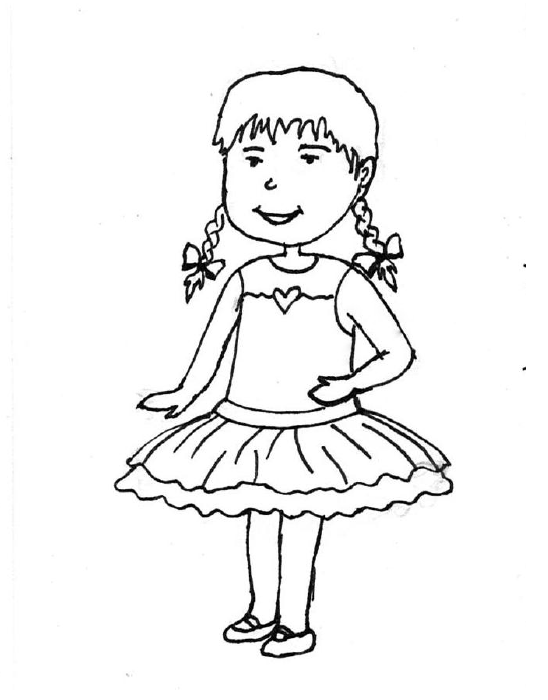** | **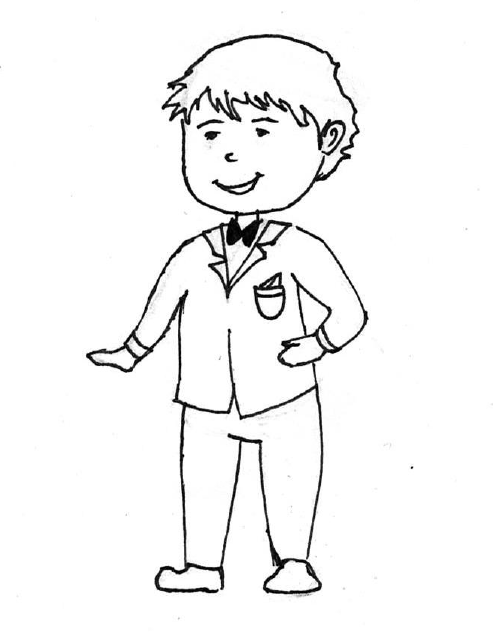** |
| **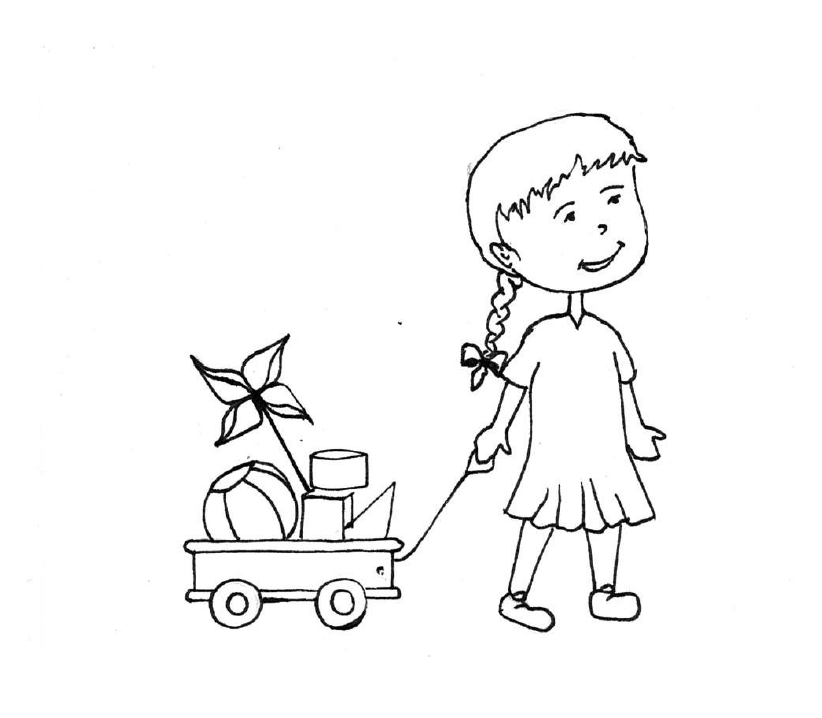** | **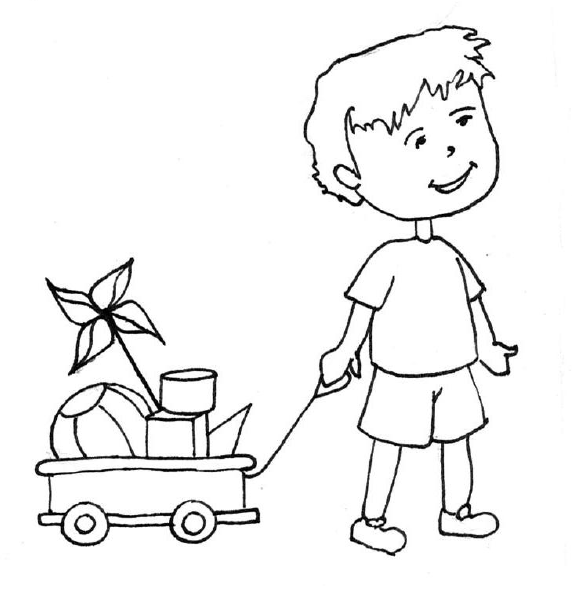** |

Supplement: S1 Appendix — (DOCX) [file pone.0290512.s001.docx]

**S2 Appendix. Happiness Collage Items**

FOR GIRLS:

**
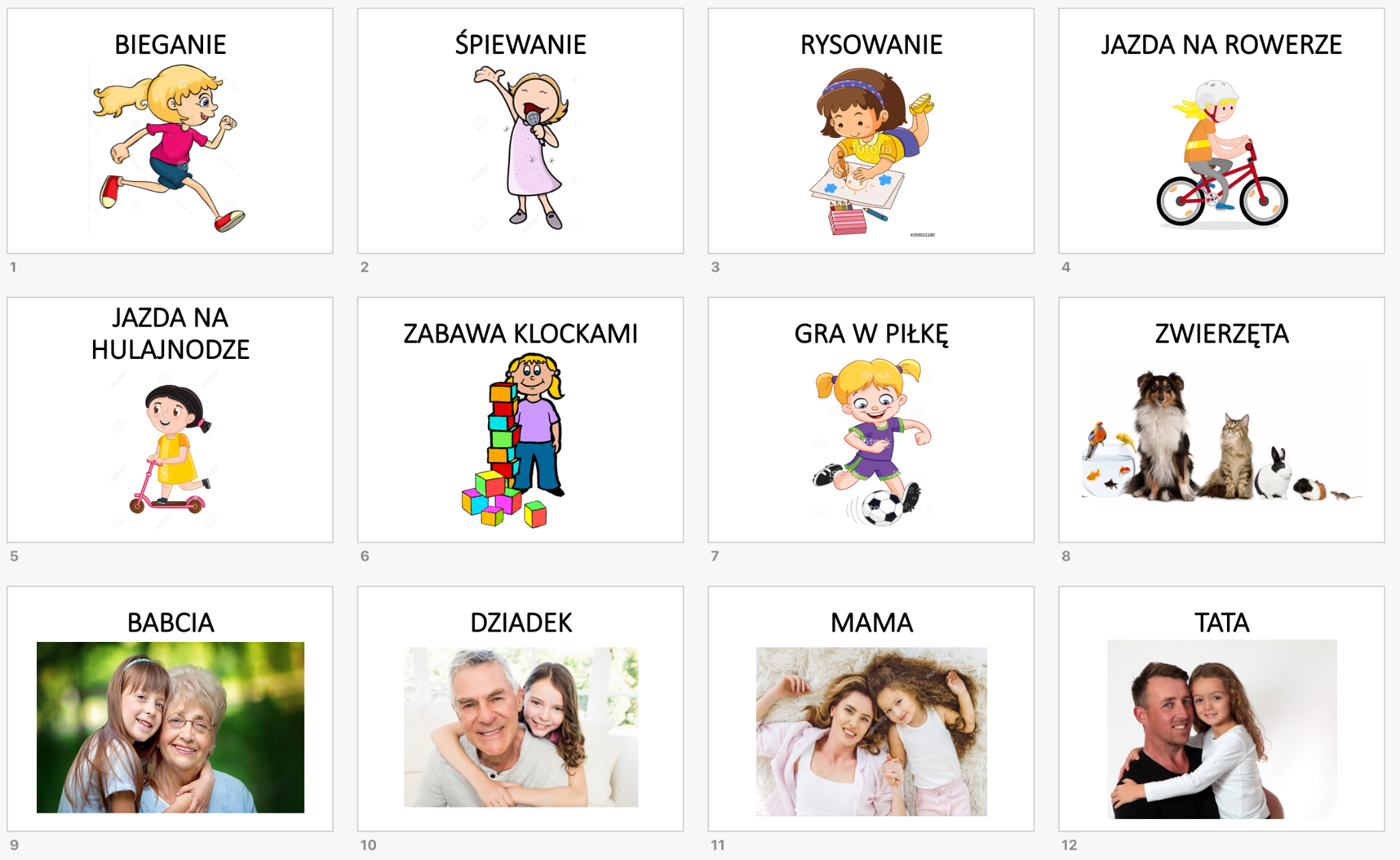
**

**
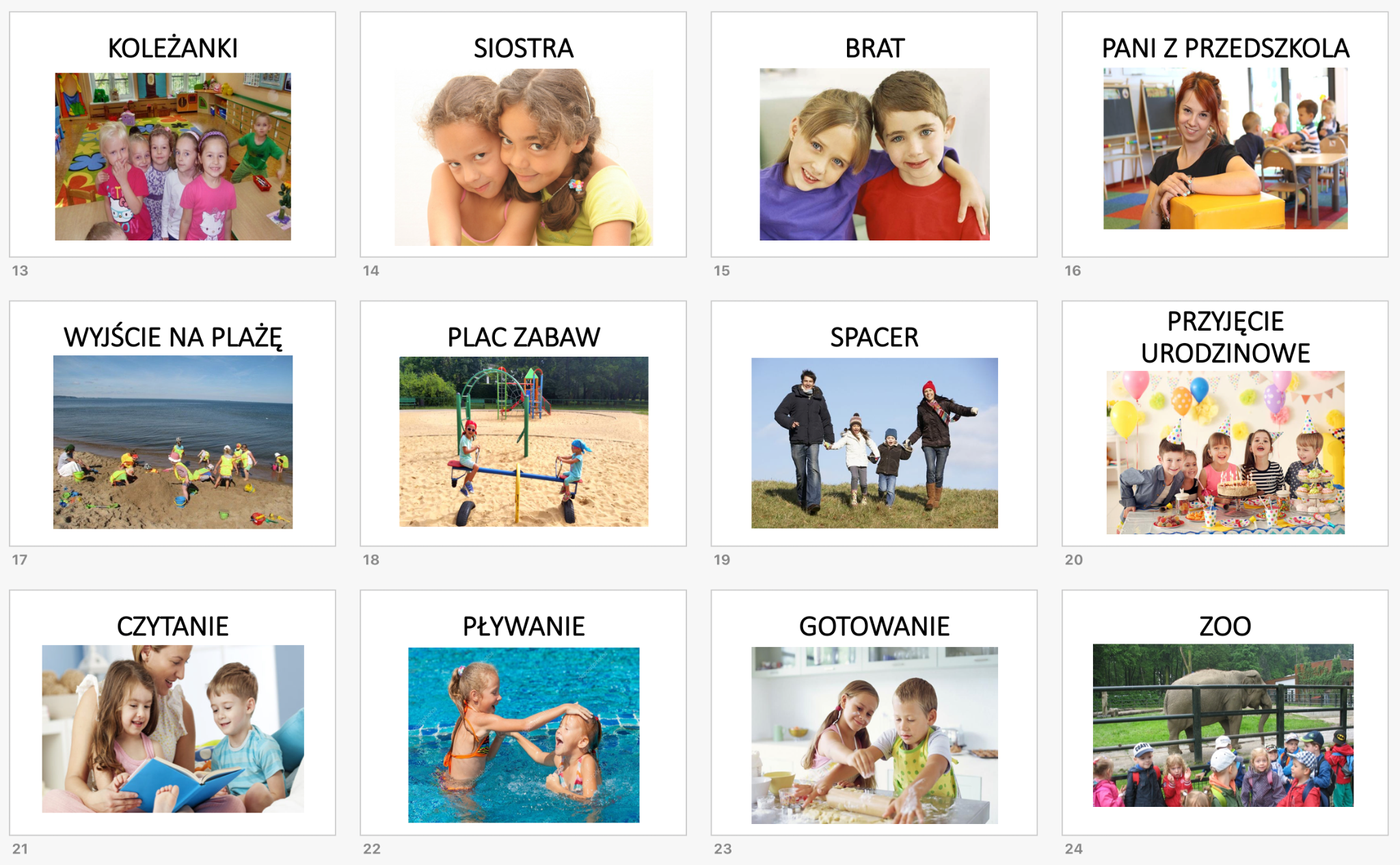
**

**
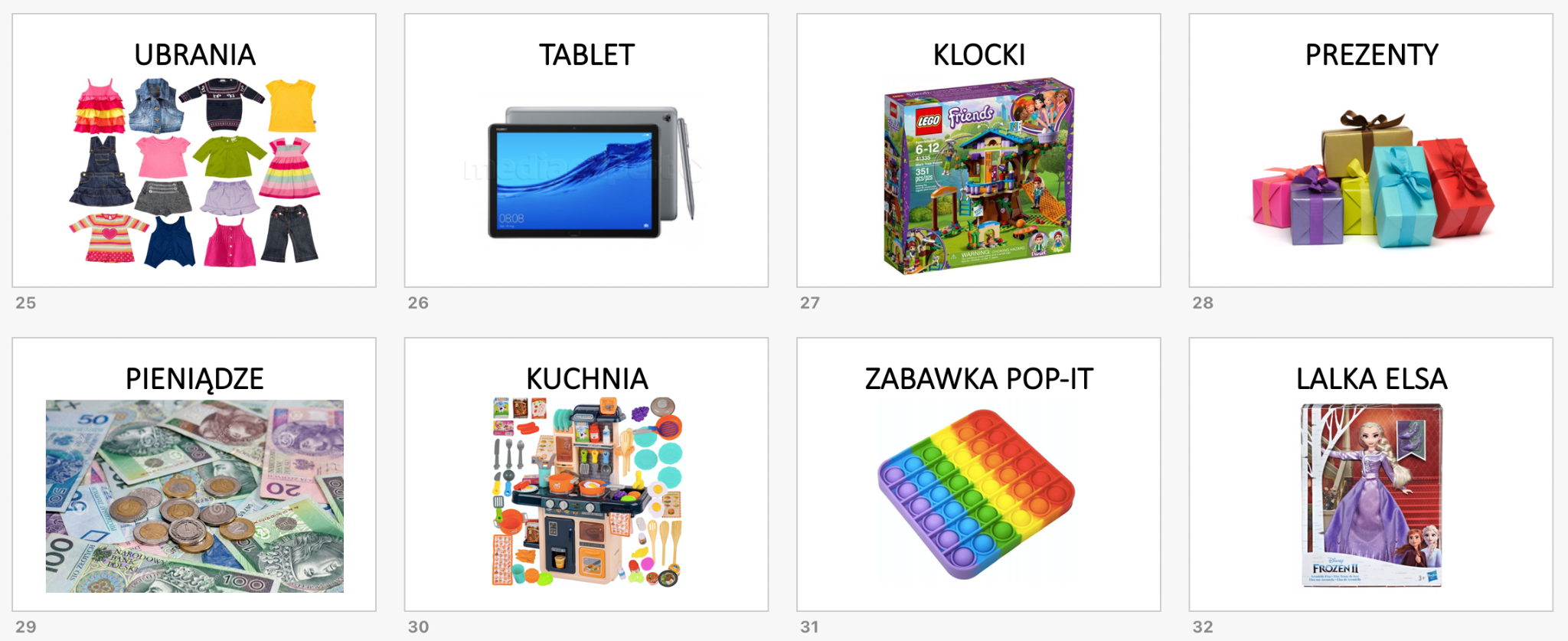
**

FOR BOYS:

**
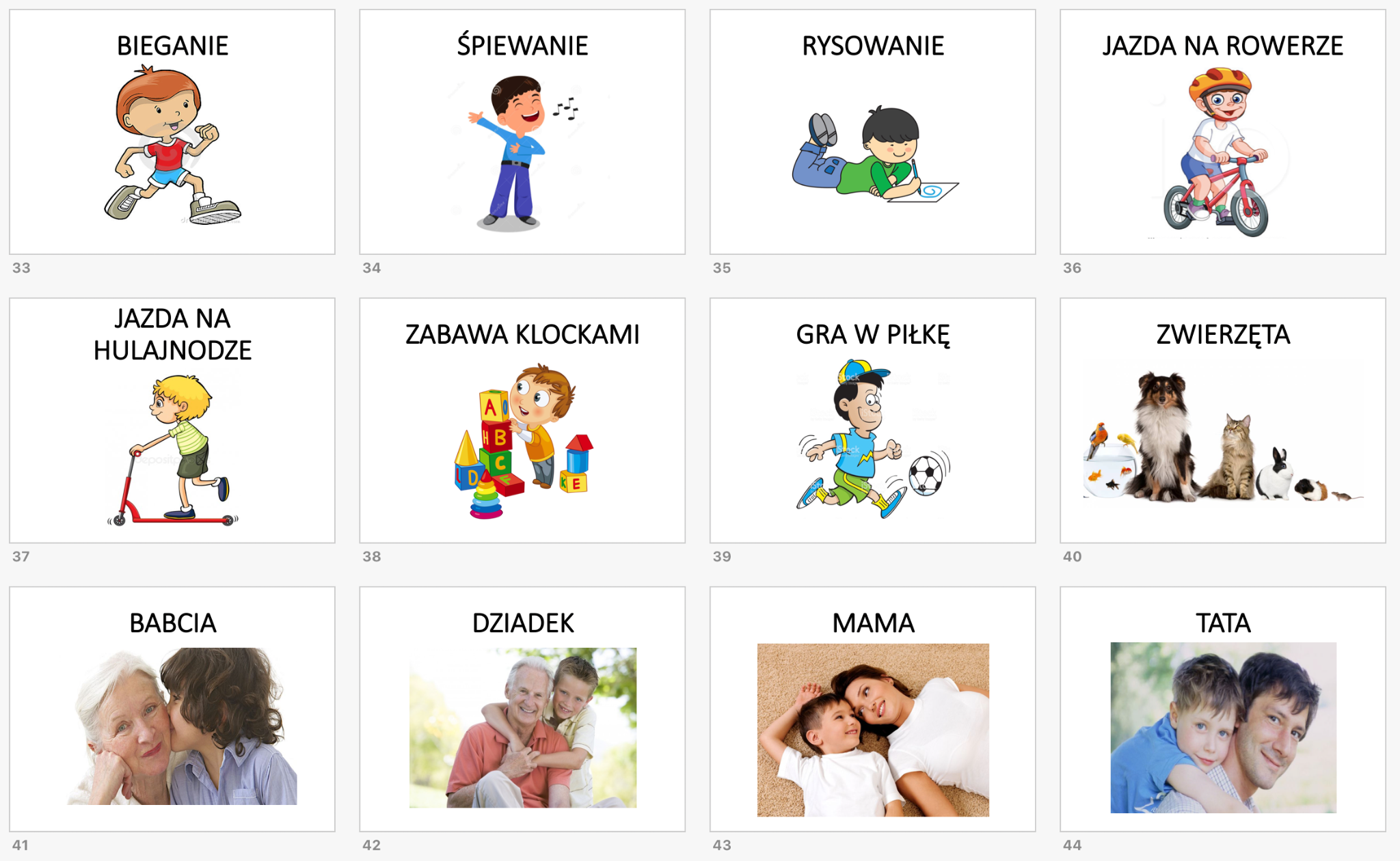
**

**
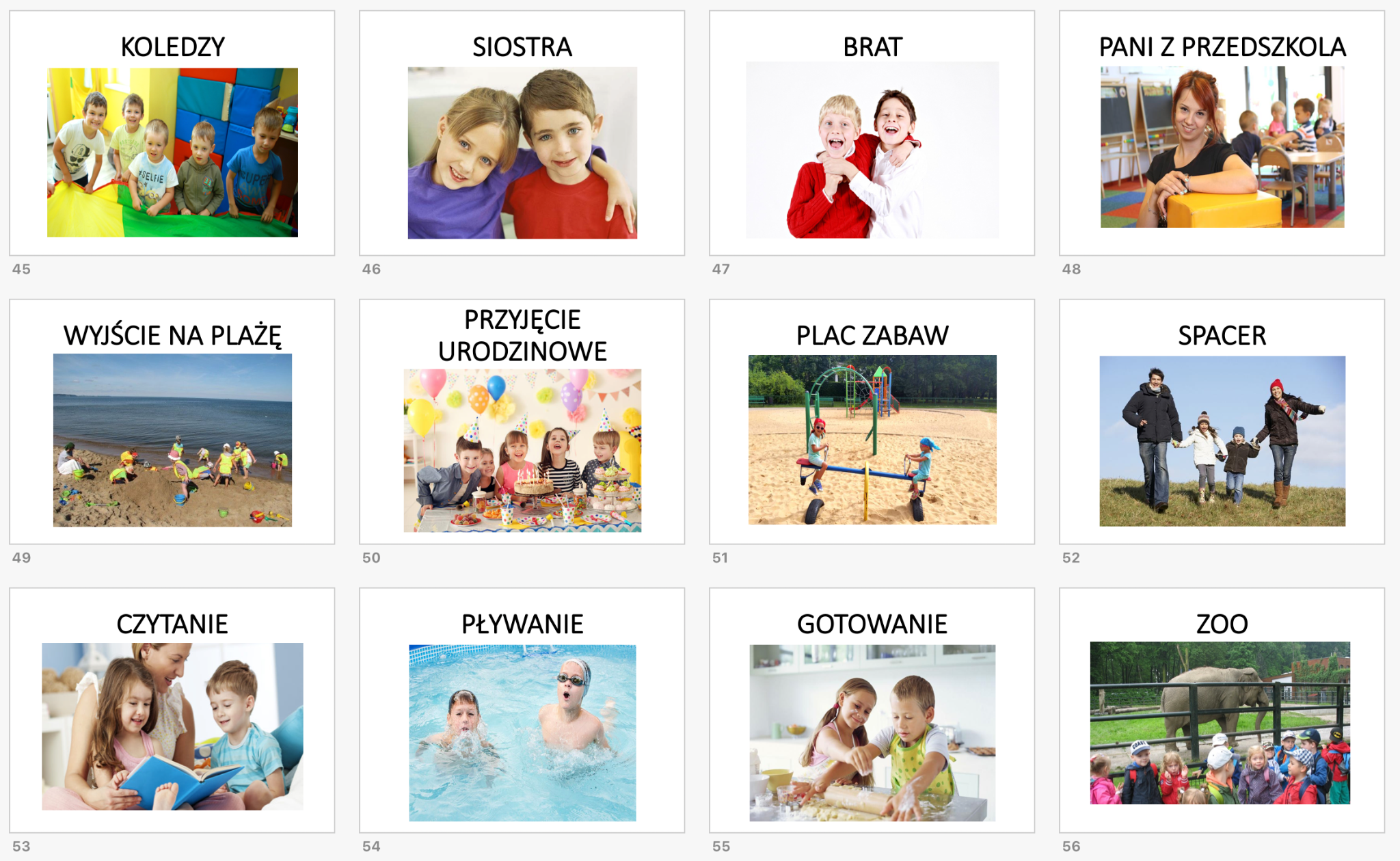
**

**
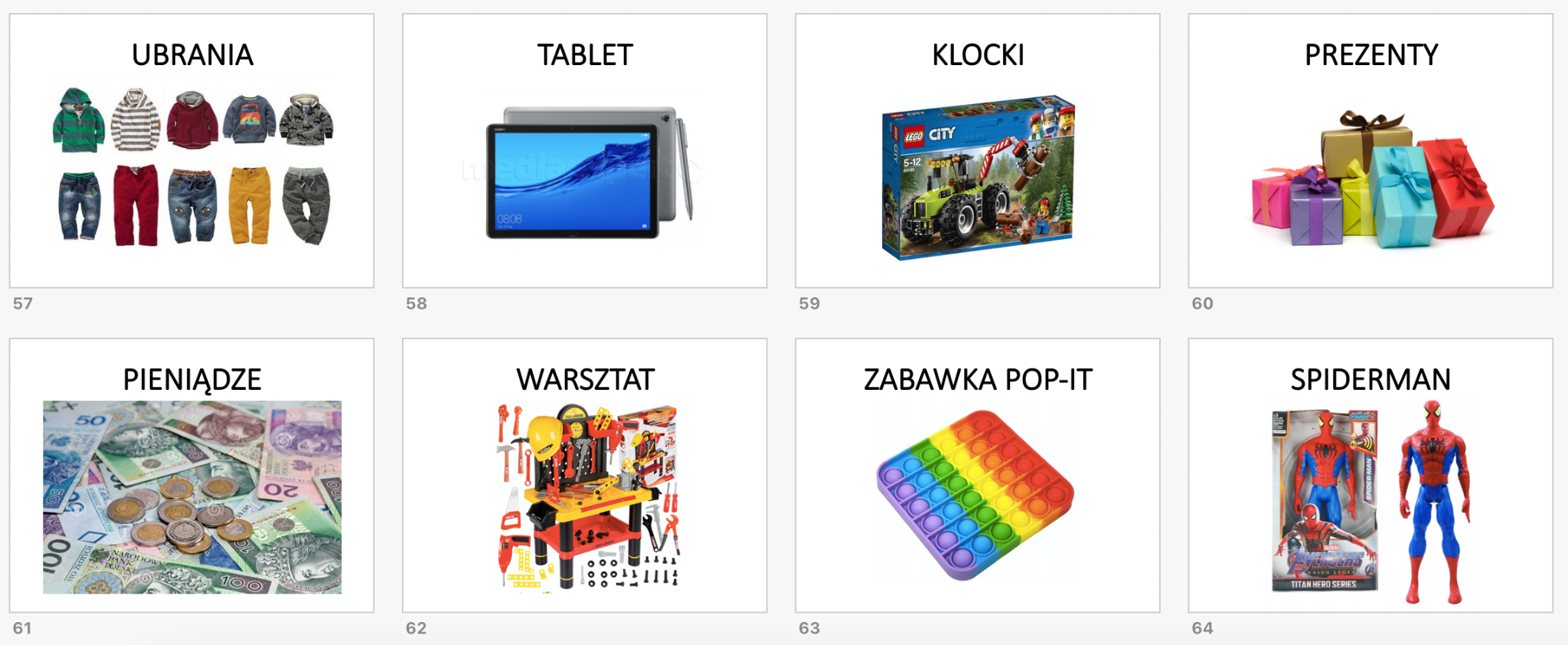
**

Supplement: S2 Appendix — (DOCX) [file pone.0290512.s002.docx]
